# A synbiotic medical food improves gut barrier function, reduces immune responses, and inhibits osteoclast activity in models of postmenopausal bone loss aligned with clinical outcomes

**DOI:** 10.1016/j.jff.2025.107114

**Published:** 2025-11-28

**Authors:** Ryan S. Green, Tyler Roy, Daniela Diaz-Infante Morales, Claire Morrow, Ryan Neilson, Eric M. Schott, Mark R. Charbonneau, Alicia E. Ballok, Katherine J. Motyl, Gerardo V. Toledo

**Affiliations:** a Sōlaria Biō, Inc, Waltham, MA 02453, USA; b Center for Molecular Medicine, MaineHealth Institute for Research, Scarborough, ME 04074, USA; c Tufts University, School of Medicine, Tufts University, Boston, MA 02111, USA; d Graduate School of Biomedical Science and Engineering, University of Maine, Orono, ME 04469, USA

**Keywords:** Osteoporosis, Probiotic, Inflammation, Menopause, Bone loss, Gut barrier

## Abstract

Over half of women above age 50 are affected by osteopenia or osteoporosis, bone-loss conditions influenced by estrogen decline, inflammation, and the intestinal microbiota. Probiotic-based interventions have shown promise in preclinical osteoporosis models. In a recent randomized, double-blind, placebo-controlled clinical trial of postmenopausal women, dietary intervention with SBD111, a synbiotic medical food combining plant-derived microbes and prebiotic fibers, reduced bone loss in women with osteopenia, elevated body mass index (BMI), and/or elevated body fat.

To investigate potential mechanisms underlying these outcomes, we examined intestinal epithelial, immune, and osteoclast responses to SBD111 in vitro. SBD111 administration improved intestinal barrier integrity, reduced immune cell cytokine secretion, and inhibited osteoclast activity. These effects align with clinically observed reductions in severe gastrointestinal symptoms and bone resorption markers. Together, these findings suggest that SBD111 modulates the gut–bone axis via barrier, immune, and antiresorptive pathways, supporting its role in maintaining skeletal health in postmenopausal women.

## Graphical abstract

**SBD111 synbiotic medical food reduces bone loss via barrier improvement, anti-inflammation, and osteoclast inhibition. (A)** SBD111 improves barrier integrity in vitro. Visualized in the left panel, inflammation (seen in red) reduces intestinal epithelial barrier function, resulting in the influx of inflammatory mediators, thereby activating the immune system. The addition of SBD111, shown in purple (right), improves barrier function, reducing the ability of inflammatory mediators to cross the epithelial barrier. **(B)** SBD111 reduces inflammatory responses of inflamed immune cells in a concentration-dependent manner. Low concentrations of SBD111 induce inflammatory responses. At higher concentrations, fewer inflammatory cytokines are secreted. **(C)** SBD111-derived components/metabolites inhibit the activity of bone-resorbing osteoclasts and reduce their ability to degrade bone. Image created in https://BioRender.com. Green, R. (2025) https://BioRender.com/agmo9s8.

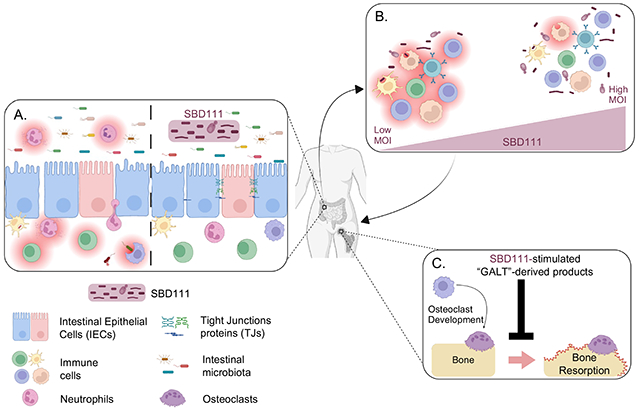

## 1. Introduction

Declining estrogen production during and after menopause is associated with systemic inflammation and rapid bone loss, and it is estimated that 50% of women over the age of 50 have osteoporosis or osteopenia (Greendale et al., 2012; Reid & McClung, 2024; Shieh et al., 2020). These women have a substantially higher risk of hip fracture than those with healthy bone mineral density (BMD), indicating a clear need to manage bone loss during and after menopause (Reid & McClung, 2024). While several pharmaceutical options exist to treat osteoporosis, including hormone therapy and bisphosphonates, adoption of these solutions is limited due to rare but serious side effects, such as increased risk of certain cancers and osteonecrosis of the jaw (Khan et al., 2023; MacLennan et al., 2004; Migliorati et al., 2010). Additionally, options to slow bone loss prior to the development of osteoporosis are limited, as these drugs are not typically prescribed for women with osteopenia.

Menopause-associated bone loss is driven by dysregulation of bone formation and resorption that is characterized by increased osteoclast activity (Møller et al., 2020; Sromová et al., 2023). Classically, osteoclastogenesis is understood to be directed by the receptor activator of nuclear factor-κB ligand (RANKL), macrophage colony stimulating factor (M-CSF), and osteoprotegerin (OPG) (Boyle et al., 2003; Takegahara et al., 2024). In addition to these factors, inflammatory cytokines, including interleukin-6 (IL-6), IL-23, and interferon gamma (IFN-γ), have been shown to modulate osteoclast activity and osteoclastogenesis (Cai et al., 2024; S. Li et al., 2024; Umur et al., 2024; Wang et al., 2025). Thus, it is now recognized that systemic inflammation plays a key role in peri- and postmenopausal bone loss (Ferbebouh et al., 2021; Zhang et al., 2022).

Substantial evidence indicates that systemic inflammation and skeletal health are regulated, in part, by the gut microbiota, a diverse community of microbes that inhabits the human gastrointestinal (GI) tract (J. Y. Li et al., 2016; Mazziotta et al., 2023; Zaiss et al., 2019). For example, gut microbes can modulate integrity of the GI epithelial barrier that serves as a primary defense against pathogens and inflammatory insults (Di Vincenzo et al., 2023a; Thoo et al., 2019). This barrier is impaired by menopause, aging, and obesity, allowing microbe-derived compounds to enter the lamina propria, induce the production of inflammatory cytokines and chemokines, and increase systemic inflammation (Di Vincenzo et al., 2023a; Shieh et al., 2020; Teixeira et al., 2012; Thevaranjan et al., 2017; Thoo et al., 2019). Certain microbial metabolites, including the short chain fatty acids butyrate and acetate, can directly inhibit these inflammatory processes and improve epithelial barrier integrity (Furusawa et al., 2013; Hosmer et al., 2024). Microbial metabolites have also been implicated as regulators of musculoskeletal health, inhibiting bone resorption by osteoclasts directly and indirectly through immunological mechanisms (Rahman et al., 2003; Tyagi et al., 2018; Zaiss et al., 2019). The structure and function of the human intestinal microbiota are substantially influenced by diet. These observations suggest that dietary interventions targeting the gut microbiota could be developed to regulate GI barrier function, systemic inflammation, and bone loss in peri- and postmenopausal women (Moles & Otaegui, 2020).

To address the unmet need of menopause-associated bone loss, we developed SBD111, a synbiotic formulation designated as a medical food for the dietary management of postmenopausal bone loss. SBD111 is comprised of four microbial strains derived from fruits and vegetables: *Levilactobacillus brevis*, *Lactiplantibacillus plantarum*, *Leuconostoc mesenteroides*, and *Pichia kudriavzevii*, as well as prebiotic fibers, oligofructose and blueberry powder, which can serve as growth substrates for these organisms (Easson et al., 2022; Lawenius et al., 2022). This synbiotic was formulated to synergistically produce acetate and to deliver a much higher concentration of viable microbes than traditional probiotic foods or supplements while including prebiotic fibers to further enhance microbial viability and function (Pandey et al., 2015). Further, SBD111 is administered in enterically-coated capsules to release microbes and prebiotic fibers directly to the site of action in the small intestine, bypassing gastric acids and enzymes (Kim et al., 2016). This strategy has been confirmed to successfully deliver viable SBD111 microbes to human participants, detectable and culturable from stool, in a recent randomized open label trial (Clinical Trial ID Number: NCT06614166) (Miller et al., 2025). Additionally, administration of SBD111 was shown to be safe and well tolerated in a double-blind, randomized, placebo-controlled 28-day administration study of healthy adult participants (Clinical Trial ID Number: NCT05206864) (Sahni et al., 2023).

The present study was motivated by findings from a recent prospective, multicenter, double-blind, randomized, placebo-controlled efficacy trial of SBD111 in 286 healthy women within six years of menopause (Clinical Trial ID Number: NCT05009875) (Schott et al., 2025). In this trial, SBD111 reduced bone loss in two prespecified populations: women with osteopenia (SBD111 test group: *N* = 43; placebo control group: *N* = 40) and those with a body mass index (BMI) ≥30 (SBD111 test group: *N* = 26; placebo control group: *N* = 16). A similar reduction was also observed in a post-hoc analysis of women with ≥40 % body fat (*N* = 130) (Schott et al., 2025). Furthermore, in women with elevated BMI, SBD111 administration was associated with decreased serum concentrations of collagen cross-linked telopeptide (CTX-1; a marker of bone degradation), suggesting inhibition of osteoclast function (Schott et al., 2025). As serum procollagen type I intact N-terminal propeptide (P1NP; a marker for bone formation) concentrations were not altered by SBD111 administration; it is likely that osteoblast function is not impacted (Schott et al., 2025). Osteopenia, elevated BMI, and elevated body fat are each associated with increased systemic inflammation (Ferbebouh et al., 2021; Festa et al., 2001; Zhang et al., 2022). As such, clinical response to SBD111 in these populations is consistent with preclinical data wherein administration of a preliminary SBD111 formulation in an ovariectomized mouse model of menopausalbone loss significantly reduced trabecular bone loss and expression of the inflammatory cytokines *Tnf* (encoding tumor necrosis factor alpha [TNF-α]) and *Il6* (encoding IL-6) within vertebral bone (Lawenius et al., 2022). Together these observations support the interpretation that immunological regulation underlies SBD111s function. Given the known links between inflammation, adiposity, and osteoclast activity, we hypothesized that SBD111 modulates the gut–immune–bone axis through epithelial, immunological, and resorptive pathways.

Here, we report that SBD111 administration improves intestinal epithelial barrier integrity, elicits concentration-dependent anti-inflammatory responses, and inhibits osteoclast activity in vitro. Each of these putative mechanisms has the potential to synergistically reduce bone loss in postmenopausal women with osteopenia or elevated BMI following administration of this synbiotic medical food.

## 2. Methods

### 2.1. Microbial strains and preparation

SBD111, a synbiotic composed of lyophilized *Levilactobacillus brevis*, *Lactiplantibacillus plantarum*, *Leuconostoc mesenteroides*, *Pichia kudriavzevii*, and prebiotic fibers, has been previously described (Easson et al., 2022; Sahni et al., 2023). Previous formulations of SBD111 (SBD111-A) included *P. fluorescens*, which was removed from the final formulation of SBD111, due to low acetate production (Lawenius et al., 2022).

SBD111 material was resuspended at a concentration of 1.48 × 10^9^ total colony forming units (CFU)/mL (CFU/mL by strain: 7.81 × 10^7^ CFU/mL *P. kudriavzevii*; 4.69 × 10^8^ CFU/mL of each: *L. brevis, L. mesenteroides*, and *L. plantarum*) and ~ 9.38 mg/mL (capsule to capsule variation within a range of 8.13–11.25 mg/mL) of each prebiotic component and in 1 X phosphate buffered saline (PBS; Catalog (CAT)# BP3991, Thermo Fisher; Waltham, MA) with regular mixing for 25 min at room temperature. *Escherichia coli* (Strain: 1100101; CAT# BAA-2471, American Type Culture Collection (ATCC), Manassas, VA) was grown overnight at 37 °C in Tryptic Soy Broth (TSB; CAT# 1.00800.0500, Merk KGAG; Darmstadt, Germany). *E. coli* was washed with 1 X PBS and resuspended in Minimum Essential Medium (MEM, phenol red-free; CAT# 51200038, Gibco^™^, Thermo Fisher; Waltham, MA), supplemented with 10 % fetal bovine serum (FBS; CAT# 16140071 Gibco^™^, Thermo Fisher; Waltham, MA) and 1 X Glutamax^™^ (CAT# 35050061, Gibco^™^, Thermo Fisher; Waltham, MA), to a concentration of 2.5 × 10^7^ CFU/mL.

### 2.2. Cells and culture conditions

Caco-2 human adenocarcinoma cells (CAT# 86010202, European Collection of Authenticated Cell Cultures, MiliporeSigma; Merck KGAG, Darmstadt, Germany) and RAW264.7 murine macrophages (CAT# TIB-71, ATCC; Manassas, VA) were cultured at 37°C and 5% CO_2_ in Dulbecco’s Modified Eagle Medium (DMEM; CAT# 10566016, Gibco^™^, Thermo Fisher; Waltham, MA) supplemented with 10 % FBS, 1X antibiotic-antimycotic (anti-anti; CAT# 15240062, Gibco^™^, Thermo Fisher; Waltham, MA), and 1X Glutamax^™^. HT29-Lucia^™^ AHR cells (CAT# ht2l-ahr, Invivogen; San Diego, CA) human adenocarcinoma cells were grown at 37°C and 5% CO_2_ in DMEM supplemented with 10% FBS, 1X Glutamax^™^, 1X anti-anti, and 100 mg/mL Zeocin^®^ (CAT# ant-zn-05, Invivogen; San Diego, CA). Cell lines were used from passage 3 to 15 for described assays.

Human peripheral blood mononuclear cells (PBMCs) were purchased from Charles River Cell Solutions (CAT# PB009C-2, Northridge, CA). Purchased PBMCs were thawed and used immediately upon receipt. PBMCs were cultured at 37°C and 5% CO_2_ in Roswell Park Memorial Institute Medium (RPMI-1640) without phenol red (CAT# 11835030, Gibco^™^, Thermo Fisher; Waltham, MA) supplemented with 10% FBS, 1X Glutamax^™^, and 12.5 mM HEPES Buffer (CAT# 15630130, Gibco^™^, Thermo Fisher; Waltham, MA). PBMCs were maintained in culture for no more than 32 h.

### 2.3. Barrier integrity assay

**Fig. 1.**
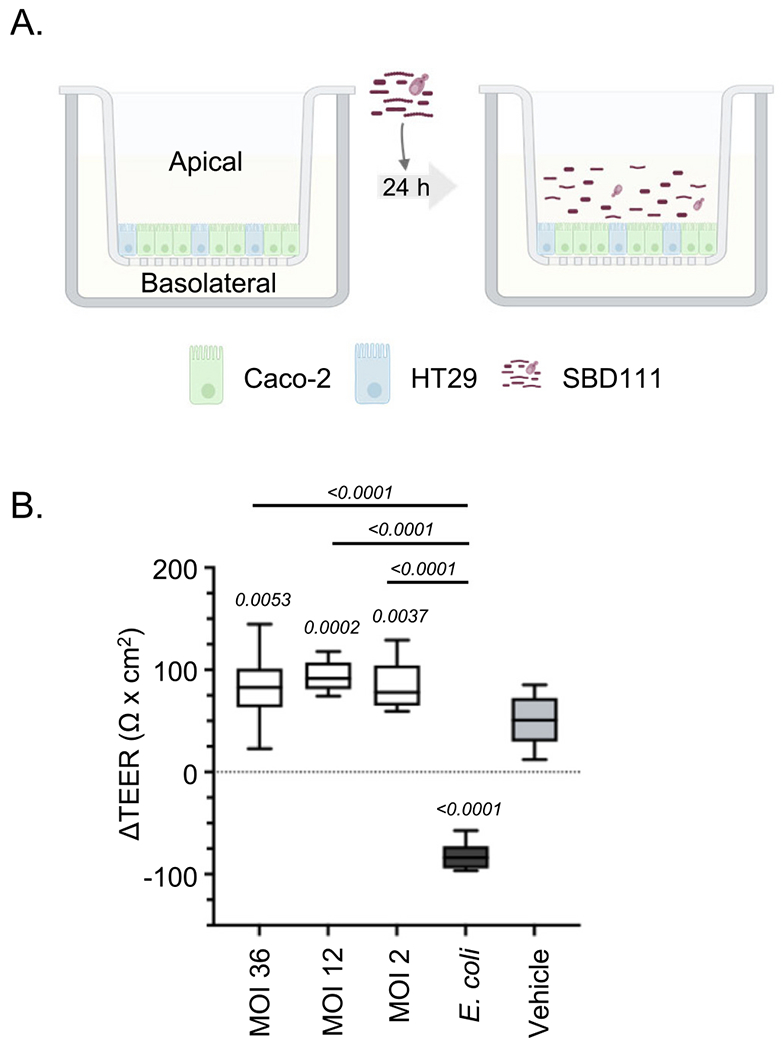
SBD111 increases barrier function (TEER) of intestinal epithelial monolayers. **(A)** Mature, polarized intestinal epithelial cell (Caco-2 and HT29) monolayers were exposed to a media control (Vehicle), disruption control (*E. coli*), or SBD111 material at a multiplicity of interaction (MOIs; microbes per human cell) of 36, 12, or 2 for 24 h. Graphic created in https://BioRender.com. Green, R. (2025) https://BioRender.com/gwva1z7. **(B)** Trans-epithelial electrical resistance (TEER) was measured before and after exposure to SBD111 (MOI 36, 12, and 2) for each monolayer, and the mean change in TEER (ΔTEER) over time was compared across the different conditions. Data presented is a single experiment that is representative of eight experiments. The central line within each box represents the mean of each condition (*N* = 12), with boxes representing interquartile range and error bars indicating the minimum and maximum values. Significance between conditions was determined by one-way ANOVA with Tukey’s HSD. *p* values are defined in italics for each statistically significant comparison (*p* < 0.05). *p* values lacking comparison bars indicate significance relative to the vehicle control.

Caco-2 and HT29 cells were cultured separately and combined at a 70:30 ratio of Caco-2 and HT29 cells, respectively (Ferraretto et al., 2018). A total of 2.5 × 10^4^ mixed Caco-2 and HT29 cells were seeded per insert on polycarbonate membrane cell culture inserts (6.5 mm diameter, 0.4 μm pore size; 3413, CAT# CLS3396-2EA Costar^™^; Corning, NY), as depicted in Fig. 1A. Each insert contained 200μL of DMEM in the apical chamber, while the basolateral chamber received 1 mL of the same medium. The plates were maintained at 37°C with 5% CO_2_. The day before the assay, the inserts were washed with 1 X PBS, and the culture medium was replaced with an antibiotic-free medium: phenol red-free MEM, supplemented with 10% FBS and 1 X Glutamax^™^, with 180μL added to the apical chamber and 1mL to the basolateral chamber.

On days 18 to 21, the trans-epithelial electrical resistance (TEER) of Caco-2-HT29 cell monolayers was measured using a voltohmmeter (Millicell^®^ ERS-2; CAT# MERS00002, EMD Millipore Corporation, Burlington, MA). A pair of electrodes (MERSSTX01, EMD Millipore Corporation, Burlington, MA) was inserted into each well to measure the resistance. TEER measurements were taken before the experiment (T0) to establish a baseline and 24 h after microbial treatment (T1) to assess the change in TEER (ΔTEER). The values were corrected for background resistance and expressed as Ω x cm^2^. The average TEER values ranged from 150 to 250 Ω x cm^2^.

Apical Caco-2:HT29 monolayers were co-incubated with 20 μL of either resuspended SBD111 material or a disruption control. Resuspended SBD111 material was diluted in MEM antibiotic-free medium to a multiplicity of interaction (MOI, microbes per human cell) of 36, 12, 2, or 0. Disruption controls were *E. coli* diluted in antibiotic free MEM to an MOI of 20 (2.5 × 10^7^ CFU/mL). The plates were incubated for 24 h at 37°C with 5% CO_2_. The apical and basolateral supernatants were collected separately for cell viability and cytokine analysis.

### 2.4. SBD111 treatments of PBMCs

Cryopreserved human PBMCs representing seven donors (four peri- and postmenopausal females, ages 51–59 years with an average age of 55 years, and three males, age 22–50 years with an average age of 37 years; additional donor information is included in Table 1) were purchased from Charles River Cell Solutions (CAT# PB009C-2, Northridge, CA). In 1.5 mL, one million cells were treated with media alone (vehicle control), 100 ng/mL of lipopolysaccharide (LPS, CAT# tlr-eblps, Invivogen; San Diego, CA) in RPMI, or resuspended SBD111 capsule contents diluted in RPMI to a MOI of 10 or 1 (Kleiveland, 2015a). Cells were incubated for 24 h at 37°C with 5% CO_2_; after which supernatants were harvested for viability and cytokine response analysis. Cell viability was determined via lactate dehydrogenase activity as per manufacturer’s instructions (CAT# G1780, Promega; Fitchburg, WI). Cytokines were analyzed via ELISA. ELISAs were performed as per manufacturer’s instructions (Thermo Fisher, Waltham, MA: TNF-α (CAT# 88–7346–88), IL-23p19 (CAT# 88–7237–88); BioLegend, San Diego, CA: IL-12p70 (CAT# 431701), IL-6 (CAT# 430501), IL-1β (CAT# 437016), IL-8/C-X-C motif ligand 8 (CXCL8) (CAT# 431501); and R&D systems, Minneapolis, MN: IFN-γ (CAT# DY285B), CXCL1 (CAT# DY275), OPG (CAT# DY805), IL-10 (CAT# DY217B)).

### 2.5. PBMC LPS challenge and SBD111 treatments

Frozen PBMCs from the healthy donors described above were incubated at 37°C with 5% CO_2_ for 30 min with either 100 ng/mL of LPS (CAT# tlr-eblps, Invivogen; San Diego, CA) in RPMI, or with media alone (unchallenged control) (Kleiveland, 2015a; Ngkelo et al., 2012). After incubations, cells were washed once with 1X PBS and resuspended in RPMI. In 1.5 mL, one million LPS-challenged or unchallenged cells were treated with an RPMI control (Vehicle) or resuspended SBD111 material diluted in RPMI to a MOI of 10 or 1. Cells were incubated for 24 h at 37° C with 5% CO_2_. Resulting supernatants were harvested and examined for viability and cytokine responses. Cell viability was determined via lactate dehydrogenase activity and cytokines were analyzed via ELISA as described above.

### 2.6. Production of GALT model supernatants

1 × 10^5^ HT29 and Caco-2 cells were seeded onto polycarbonate membrane cell culture inserts (12 mm diameter, 0.4 μm pore size; CAT#3401, Costar; Corning, NY) at a 70:30 ratio of Caco-2 to HT29 cells in 0.5 mL of DMEM (Ferraretto et al., 2018). 1.5 mL of DMEM was added to each well. After 1 week of culture, media was replaced with αMEM without phenol red (CAT#41061029, Gibco^™^, Thermo Fisher; Waltham, MA) supplemented with 10 % charcoal-stripped FBS (CAT# F6765, MiliporeSigma; Merck KGAG, Darmstadt, Germany) and 1X Glutamax^™^ for an additional 11–14 days; during which media continued to be replaced every 48 h. Cryopreserved PBMCs from a peri-, postmenopausal female donor (age 58 years) were thawed and resuspended in αMEM supplemented with 10% charcoal-stripped FBS and 1X Glutamax^™^. One million PBMCs were added to the basolateral chamber of each epithelial cell monolayer containing well to produce a gut-associated lymphoid tissue (GALT) model as described previously (Kleiveland, 2015b; Korsten et al., 2023). The apical side of each GALT model was incubated with an αMEM control (vehicle), SBD111 material, diluted to an MOI of 10 or 2 (relative to epithelial barrier), or LPS, diluted to 500 ng/mL in αMEM supplemented with 10 % charcoal-stripped FBS and 1 X Glutamax^™^. Cells were incubated for 24 h at 37°C with 5% CO_2_; after which microbe free, basolateral supernatants were harvested for viability, osteoclastogenesis modeling, and cytokine response analysis. Supernatants harvested for osteoclastogenesis modeling were filter sterilized with 0.2 μm cellulose-acetate syringe filters. Cells were harvested for RANKL and OPG gene expression analysis via qRT-PCR.

### 2.7. RAW264.7 cell osteoclast model

2.5 × 10^4^ RAW264.7 macrophages in 140 μL of DMEM were incubated for 7 days at 37°C with 5% CO_2_ in DMEM containing 50 ng/mL of RANKL (Quach et al., 2019). Cells were treated with 60 μL of undiluted basolateral supernatants from the GALT models, described above. Alternatively, RAW264.7 macrophages were treated with SBD111-conditioned αMEM, which was produced by culturing resuspended SBD111 material at a concentration of 6.67 × 10^5^ or 1.33 × 10^5^ CFU/mL (MOI 10 and 2 equivalent concentrations) in αMEM (supplemented with 10 % charcoal-stripped FBS and 1 X Glutamax^™^) for 24 h at 37°C and 5% CO_2_. The resulting conditioned supernatant was filter sterilized with 0.2 μm cellulose-acetate syringe filters prior to incubation with RAW264.7 cells. After one week, supernatants were harvested to assay lactate dehydrogenase activity as per manufacturer’s instructions to determine viability (CAT# G1780, Promega; Fitchburg, WI). Cells were analyzed for tartrate-resistant acid phosphatase (TRAP) activity to quantify osteoclast differentiation as per manufacturer’s instructions (CAT# MK301, Takara; San Jose, CA).

### 2.8. Human osteoclast model

Human PBMCs were isolated from whole blood samples collected from four female patients ages (65–87) mean age of 74 into EDTA tubes. Blood samples were transferred into a 50 mL centrifuge tube (CAT# 21008–690, Corning; Corning, NY) and an equal volume of sterile 1X PBS Buffer with 2% FBS (CAT# 07905, Stemcell^™^ Technologies; Cambridge, MA) was added. PBMCs were separated using Sepmate^™^ tubes (CAT# 85460, Stemcell^™^ Technologies; Cambridge, MA) with 15 mL Ficoll-Paque^™^ Plus (CAT# 95021–205, Cytiva; Marlborough, MA) and centrifuged for 20 min at 1200 *x g*. Isolated PBMCs were decanted into a new 50 mL centrifuge tube washed with 40 mL of 1 X PBS with Buffer 2% FBS and centrifuged at 300 *x g* for 10 min. Following centrifugation, the supernatant was discarded, and wash steps were repeated for a second time. Finally, the PBMC pellet was triturated and suspended in plating media on ice and counted manually with a hemocytometer.

#### 2.8.1. Human osteoclast culture

PBMCs isolated from human blood samples were seeded on bone chips (CAT# DT-1BON1000–96, Immunodiagnostic Systems, East Boldon, UK; Boneslices.com) at a density of 1.25 × 10^6^ cells/cm^2^ which is equivalent to 4 × 10^5^ cells/well on a 96 well plate. For the culture, PBMCs and bone chips are suspended in a 2:1 ratio of osteoclastogenic media and basolateral GALT supernatant, as produced above. The osteoclastogenic media is comprised of αMEM without phenol red (CAT#41061029, Gibco^™^, Thermo Fisher; Waltham, MA) supplemented with 10% charcoal-stripped FBS (CAT# F6765-500ML, MiliporeSigma, Merck KGAG, Darmstadt, Germany), 1X Pen-Strep (CAT# 15140122, Thermo Fisher; Waltham, MA), RANKL (CAT# 390-TN-010/CF, R&D systems, Minneapolis, MN), M-CSF (CAT# 216-MC-010, R&D systems, Minneapolis, MN). The media mixture resulted in final concentrations of 50 ng/mL RANKL and 25 ng/mL M-CSF. Once PBMCs were seeded, they were cultured for 14 days at 37°C with media changes every 48–72 h. Finally, cells were fixed using 2.5% glutaraldehyde (CAT# 16220, Electron Microscopy Sciences; Hatfield, PA) on day 14 after conditioned media was collected.

#### 2.8.2. Quantifying osteoclast differentiation and activity

Media was collected from day 12 of the osteoclast culture to measure CTX-1 released into media as a measure of osteoclast resorptive activity. Levels of CTX-1 in each well of treated media were analyzed using CrossLaps^®^ for Culture (CTX—I) ELISA (CAT# AC-07F1, Immunodiagnostic Systems, East Boldon, UK).

On day 14, cells were fixed with 2.5% glutaraldehyde, and the bone chips were stained for TRAP positive cells using a TRAP kit (CAT # 387 A-1KT, MiliporeSigma, Merck KGAG, Darmstadt, Germany) for 1 h and 45 min. Stained bone chips were then imaged using the Keyence BZ-X800 microscope at 10× magnification. TRAP positive osteoclasts were quantified, and total area was analyzed using the NOISe machine learning algorithm (Kumar et al., 2024).

### 2.9. Statistical analyses

**Fig. 2.**
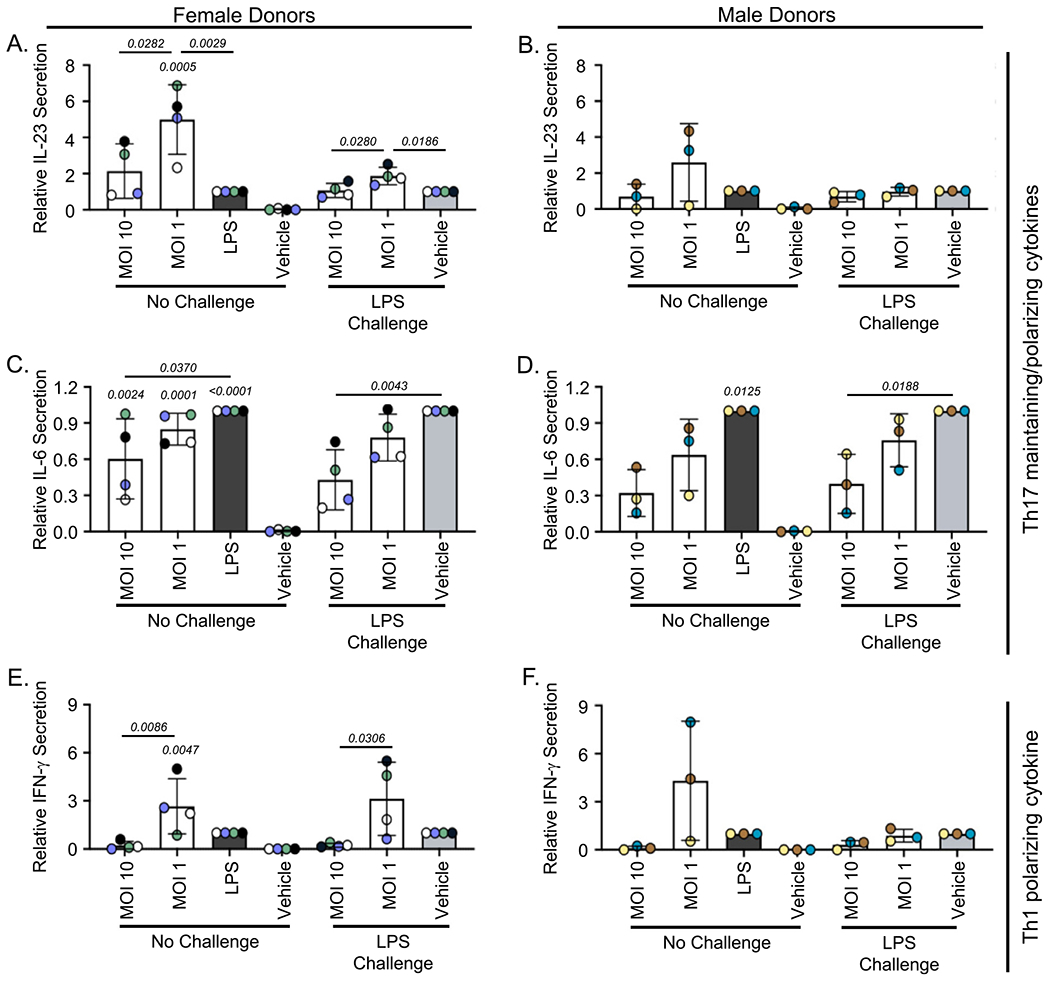
SBD111 induces concentration-dependent alterations in T cell polarizing cytokine secretion by human PBMCs both at baseline and after inflammatory challenge. Human peripheral blood mononuclear cells (PBMCs) from four peri- and postmenopausal females (**A, C**, and **E**) and three males (**B, D**, and **F**) were pretreated with media or 100 ng/mL of LPS for 30 min to induce inflammatory responses. After pretreatment, PBMCs were exposed to a vehicle control (media), lipopolysaccharide (LPS; a stimulatory control), or SBD111 material at an MOI of 10 or 1 for 24 h. After the incubation, the secretion of T cell polarizing cytokines elicited by SBD111 were determined via ELISA. Th17 maintaining/polarizing cytokines: (**A** and **B**) IL-23, (**C** and **D**) IL-6; Th1 polarizing cytokines: and (**E** and **F**) IFN-γ. Data was normalized to LPS (inflammation naïve conditions) or LPS-challenged, vehicle-treated controls (LPS challenge) to compare across donors and experiments. Columns indicate the mean ± standard deviation (SD) for all donors of the same sex (Female donors *N* = 4. Male donors *N* = 3). Points indicate the mean response of an individual donor and are color coded by donor (Females: Donor 1 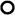; Donor 2 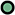; Donor 3 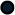; Donor 4 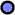. Males: Donor 5 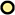; Donor 6 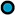; Donor 7 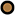). Significance between conditions within challenge status was determined by one-way ANOVA with Tukey’s HSD and *p* values are presented for each significant relationship (*p* < 0.05). *p* values without comparison bars indicate significance relative to the vehicle control for inflammation naïve conditions.

**Fig. 3.**
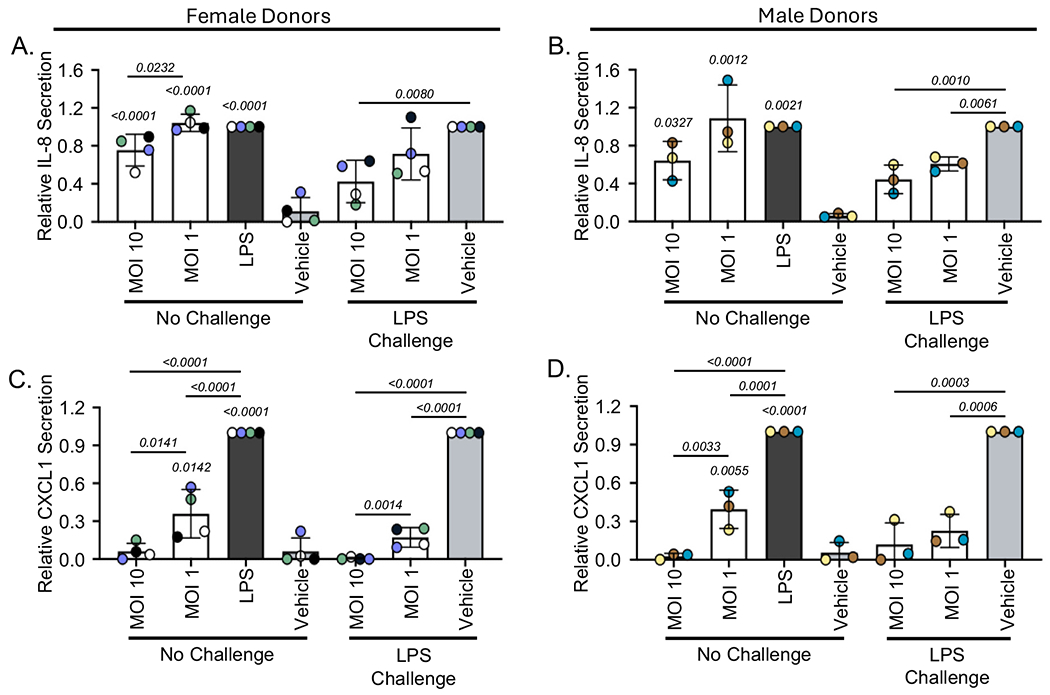
SBD111 induces concentration-dependent reductions in neutrophil-chemoattractant secretion human PBMCs both at baseline and after inflammatory challenge. Human peripheral blood mononuclear cells (PBMCs) from four peri- and postmenopausal females (**A** and **C**) and three males (**B** and **D**) were pretreated with media or LPS (100 ng/mL) to stimulate inflammatory responses. Subsequently, cells were exposed to a vehicle control (media), lipopolysaccharide (LPS; a stimulatory control), or SBD111 material at an MOI of 10 or 1 for 24 h. After the incubation, neutrophil-attractive chemokine secretion by PBMCs was determined by ELISA (**A** and **B**: IL-8; **C** and **D**: CXCL1.) and normalized to compare across donors and experiments. Inflammation naïve conditions were normalized to LPS, while inflammatory challenge conditions were normalized to LPS-challenged, vehicle-treated controls. Columns indicate the mean ± standard deviation (SD) for all donors of the same sex (Female donors N = 4. Male donors N = 3). Points indicate the mean response of an individual donor and are color coded by donor (Females: Donor 1 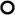; Donor 2 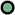; Donor 3 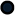; Donor 4 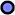. Males: Donor 5 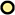; Donor 6 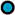; Donor 7 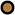). Significance between conditions within challenge status was determined by one-way ANOVA with Tukey’s HSD and *p* values are presented for each significant relationship (*p* < 0.05). *p* values without comparison bars indicate significance relative to the vehicle control for inflammation naïve conditions.

Data were analyzed using the Prism graphing and analysis software (GraphPad Software, Boston, MA). Normality was determined by Shapiro-Wilk test, while variance was determined for unnormalized data by Brown-Forsythe test. When assumptions were met, one-way ANOVA with Tukey’s HSD, to correct for multiple comparisons, was used. When assumptions were not met, Brown-Forsythe Test and Welch’s ANOVA with Dunnett’s T3 multiple comparisons post hoc analysis was used. When normalized data was analyzed by challenge state, as in Fig. 2, Fig. 3, and Supplementary Fig. 4, individual statistical analysis was performed for each challenge state (No challenge or LPS challenge). When multiple donors were examined individually, as in Supplementary Fig. 2, 3, and 5, statistical analysis was performed separately for each donor.

## 3. Results

### 3.1. SBD111 administration improves intestinal barrier integrity

SBD111 administration was associated with reduced severe gastrointestinal symptoms in post-menopausal women, suggesting direct effects on cells of the GI tract. Additionally, SBD111 is administered orally in an enterically-coated capsule format that releases its contents upon neutrality in the ileum, protecting the contained microbes and prebiotic fibers from digestion in the stomach and duodenum (Kim et al., 2016). This results in initial interactions with the human body at the intestinal epithelium. As such, we prioritized investigating the effects of SBD111 on intestinal epithelial cells. To examine whether SBD111 affects barrier integrity, intestinal epithelial cell monolayers were established on semi-permeable membranes, as illustrated in Fig. 1A. These monolayers were exposed to a media control (Vehicle), or to SBD111 capsule contents, including prebiotic fibers. Entire capsule contents were administered in this model to represent capsule release and the interconnected function of all components. For example, the prebiotic components function as a microbial nutrient source for SBD111, improving microbial viability, growth, and beneficial metabolite production, with possible direct immunological activity. Additionally, we previously showed that the microbial consortium present in SBD111 synergistically produces anti-inflammatory compounds relative to its individual components, supporting the rationale for examination of complete capsule contents (Lawenius et al., 2022). The ratios of microbes to epithelial cells utilized in this assay were selected to recapitulate the range of exposure expected to occur in the human gut. SBD111 is administered at 5 × 10^10^ CFU per dose and released within the ileum, which has a surface area of ~18 m^2^, and human colonic biopsies and murine jejunum have been reported to contain 4.2 × 10^6^ and 1.9 × 10^7^ cells/cm^2^, respectively (Abbott et al., 2013; Cheng et al., 1984; Cheng & Bjerknes, 1983; Helander & Fändriks, 2014). Given these assumptions, SBD111 exposure to intestinal epithelial cells is expected to occur at an MOI range of 1.4–6.2 in human trials. Based on this, a MOI of 2 was selected to represent a uniform distribution of SBD111. However, capsule contents are released in a localized area as a bolus. To model the likely higher MOI that occurs near the site of capsule release, MOIs of 12 and 36 were also used. *E. coli* was used as a barrier disruption control, as it has been demonstrated to damage barrier integrity in vitro (Yuan et al., 2020). The effect of SBD111 on intestinal barrier function was assessed by changes in TEER across the monolayer at 0 and 24 h incubation. As shown in Fig. 1B, all tested MOIs — 36, 12, and 2 — resulted in a significant increase in TEER compared to the vehicle control, though there were no significant differences between these groups. *E. coli* significantly reduced TEER as expected.

Intestinal epithelial cell immune responses toward SBD111 were examined through the secretion of IL-8 and CXCL1, chemokines associated with intestinal inflammation and neutrophil recruitment (Supplementary Fig. 1) (Capucetti et al., 2020). SBD111 administration did not significantly increase epithelial chemokine secretion compared to the vehicle control. By contrast, secretion of both chemokines was significantly increased by *E. coli* application, as expected.

### 3.2. SBD111 elicits concentration-dependent anti-inflammatory responses from human immune cells

To explore how SBD111 may modulate systemic inflammation to benefit populations with osteopenia or high BMI, we treated human peripheral blood mononuclear cells (PBMCs) with increasing concentrations of this synbiotic, a technique commonly used to examine probiotic-immune interactions (Belguesmia et al., 2019; Hidalgo-Cantabrana et al., 2018; Schott et al., 2025; Sornkayasit et al., 2024). This approach was motivated by the known association between elevated BMI, bone loss, and inflammation (Ferbebouh et al., 2021; Festa et al., 2001; Zhang et al., 2022).

Cryopreserved human PBMCs isolated from four peri- or postmenopausal female donors as well as three male donors were treated with a media control (vehicle), a stimulatory control (LPS), or SBD111 material at a total MOI of 10 or 1. It has been established that some immune cells directly monitor the intestinal lumen and that specialized cells translocate bacteria into immunological compartments (Da Silva et al., 2017; Farache et al., 2013). For this reason, an MOI of 1 was chosen to represent the low abundance stimulation experienced by intestinal immune cells in a healthy gut. Additionally, a higher MOI of 10 was included to reflect the increased bacterial translocation into tissues that accompanies the menopause transition and high BMI/body fat, a population that benefits from SBD111 administration (Shieh et al., 2020; Teixeira et al., 2012; Thevaranjan et al., 2017). After 24 h, cytokine responses were analyzed. As expected, we found that SBD111 stimulated inflammatory cytokine secretion in inflammation-naïve PBMCs (Fig. 2, Fig. 3, Supplementary Fig. 2, and Supplementary Fig. 4). As PBMCs protect the body from microbial challenge, this response is anticipated for probiotic microbes (Hua et al., 2010; Ren et al., 2019). However, an SBD111 concentration-dependent reduction in cytokine secretion was observed for many inflammatory cytokines. Across the four female donors, SBD111 administration at an MOI of 10 resulted in significantly reduced secretion of IL-23, IFN-γ, IL-8, and CXCL1 relative to an MOI of 1 (Fig. 2A and E; Fig. 3A and C). These cytokines are important inflammatory signals that function in inflammatory T cell polarization/maintenance (Fig. 2A and E; IL-23 and IFN-γ) and neutrophil chemoattraction (Fig. 3A and C; IL-8 and CXCL1) (Capucetti et al., 2020; Cui et al., 2025; Muranski & Restifo, 2013). For CXCL1, IL-23, and IFN-γ, SBD111 administration at an MOI of 10 did not result in a significant increase compared to the media control. The three male donor-derived PBMCs exhibited similar CXCL1 secretion patterns (Fig. 3D) relative to the female donors. IL-23, IFN-γ, and IL-8 secretion exhibited the same trends as the female donors but were not statistically significant relative to MOI 1 (Fig. 2B and F; Fig. 3B). While donor variation was seen across cytokine responses, each donor trended similarly, as indicated by donor-specific coloration, defined in Table 1, in Fig. 2, and Fig. 3 and shown as absolute cytokine secretion by donor in Supplementary Fig. 2 and 3. Other examined cytokines included TNF-α and IL-1β, which were secreted in response to SBD111 (Supplementary Fig. 4A, B, C, and D). IL-12, a Th1 cytokine mirrored the trends seen for IFN-γ, but was highly donor-dependent (Supplementary Fig. 2D and 3D). The anti-inflammatory factor, IL-10, was secreted variably between donors and was significantly reduced by SBD111 in the inflammatory challenge model (Supplementary Fig. 4E and F).

To model the effects of SBD111 administration under conditions more representative of the systemic inflammation observed in women with osteopenia and BMI 30+ post-menopause, an LPS challenge model was used (Kleiveland, 2015a; Ngkelo et al., 2012). PBMCs isolated from the same four female and three male donors were briefly treated with LPS, after which the LPS was removed and PBMCs were exposed to a media control (vehicle) or SBD111 material diluted to a total MOI of 10 or 1. Brief LPS challenge alone did not robustly induce Th1 cytokine secretion (IFN-γ and IL-12), consistent with prior reports (Supplemental Fig. 3C and D) (Janský et al., 2003). In contrast to the inflammation-naïve responses, SBD111 significantly reduced IL-6, IL-8, and CXCL1 secretion in a concentration-dependent manner relative to the LPS-challenged vehicle control in female and male PBMCs (Fig. 2A and B; Fig. 3). When IL-23 and IFN-γ were examined, both trended similarly to the unchallenged responses for females, however, neither IL-23 nor IFN-γ secretion was significantly reduced by SBD111 at MOI 10, relative to the vehicle control (Fig. 2B and F). Additionally, during stimulatory conditions, male PBMCs exhibited different trends from female donors for IL-23 and IFN-γ. This is not unexpected, as important sources of these cytokines (dendritic cells, monocytes, and T cells) differ between sexes (Pal et al., 2024; Scotland et al., 2011; White et al., 2022).

### 3.3. Basolateral-conditioned media from apical SBD111 GALT exposure reduces osteoclast activity

Orally administered microbes can exert potent immune effects locally in the GI tract that translate into systemic changes, including effects on osteoclastogenesis (Di Vincenzo et al., 2023a; Zaiss et al., 2019). Given the significant reductions in bone loss and serum CTX observed in participants with BMI ≥30 in the clinical trial, we sought to investigate whether SBD111 alters osteoclastogenic signaling via its interaction with intestinal immune tissues (gut-associated lymphatic tissue: GALT) in conditions mimicking an intact gut epithelial barrier (Kleiveland, 2015b; Korsten et al., 2023). This model contains many of the relevant cell types found within GALT producing a more physiologically relevant model than individual cell models and has been used to dissect mechanism of action in vitro.

**Fig. 4.**
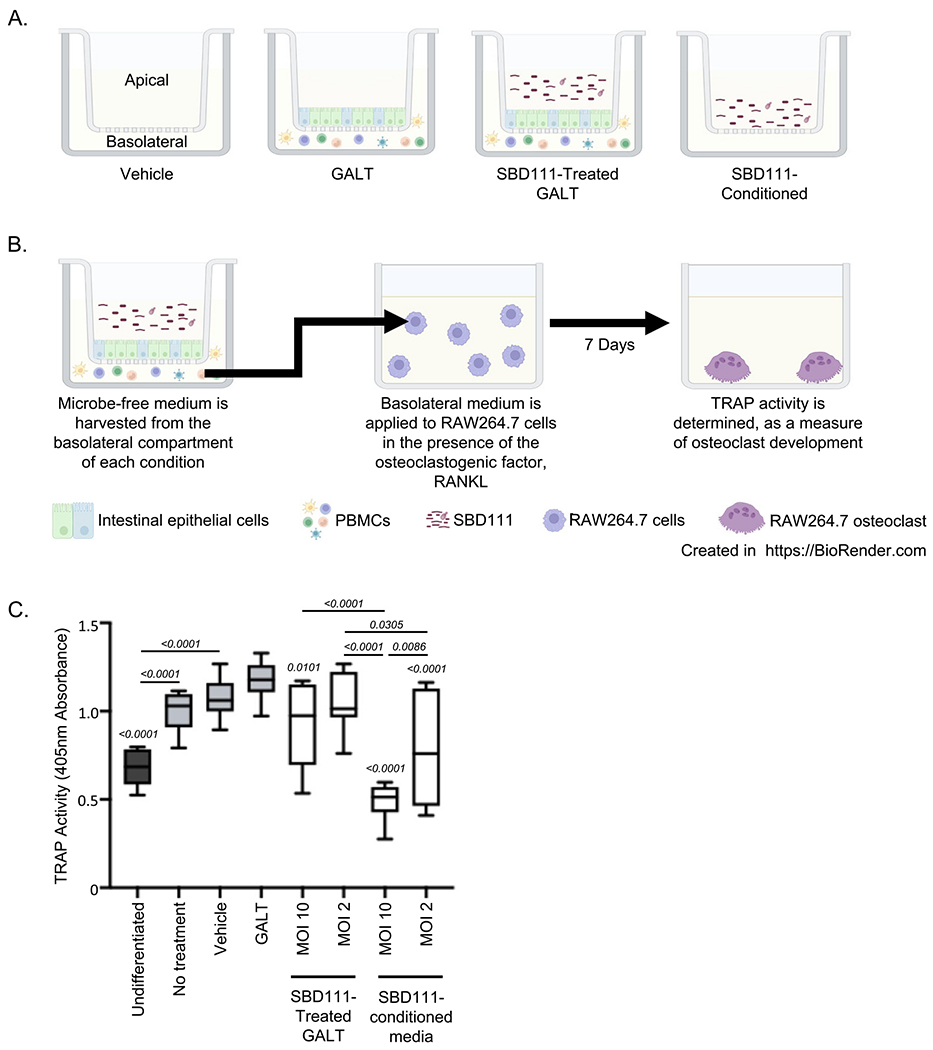
SBD111 reduces osteoclastogenesis in murine RAW264.7 models. Gut-associated lymphoid tissue (GALT) models were established containing polarized intestinal epithelial cell (Caco-2 and HT29) monolayers on cell culture inserts. Human PBMCs were added to the basolateral compartment. A media control (vehicle) or SBD111 material at MOIs of 10 or 2 were added to the apical compartment of the GALT model for 24 h. For SBD111-conditioned media without human cells, concentrations equivalent to an MOI of 10 or 2 were used. (**A**) A graphical representation of the treatments that were used in this experiment. (**B**) A workflow illustration depicting basolateral supernatants were harvested from each GALT condition and applied to RAW264.7 cells for seven days to examine the effects of each GALT treatment on osteoclastogenesis. Graphics created in https://BioRender.com.Green, R. (2025) https://BioRender.com/vnpczon. (**C**) RAW264.7 cells were treated with media (Undifferentiated) or 50 ng/mL of receptor activator of nuclear factor-kB (NF-kB) ligand (RANKL). RANKL-treated cells were further treated with RAW264.7 media (No treatment), GALT model media (Vehicle), media control-treated GALT-conditioned media (GALT), SBD111-treated GALT-conditioned media (MOI 2 or 10), or SBD111-conditioned media without human GALT (MOI 10 or 2). RAW264.7 cells were cultured for seven days. After seven days, osteoclastogenesis was determined by measuring tartrate-resistant acid phosphatase (TRAP) activity. Results are presented as the mean of 8–14 data points for each condition (14 replicates – Undifferentiated, GALT, and LPS; 13 Replicates – Differentiated and Vehicle; and 8 conditions for SBD111-treated GALT and SBD111 conditioned media), with boxes representing interquartile range and error bars indicating the minimum and maximum values. Significance between conditions was determined by oneway ANOVA with Tukey’s HSD. Individual *p* values are indicated for significant comparisons (*p* < 0.05). *p* values comparison bars indicate significance relative to the GALT control.

Briefly, intestinal epithelial monolayers were established on cell culture inserts as illustrated in Fig. 1A. PBMCs were added to the basolateral chamber to represent the immune components of GALT, while SBD111 was applied to the apical compartment, representing the intestinal lumen, as illustrated in Fig. 4A. After 24 h, the basolateral supernatant (PBMC and intestinal epithelial cell-conditioned supernatant lacking direct microbial exposure) was harvested to examine the osteoclastogenic potential of secreted factors, and cellular RNA was isolated to quantify gene expression. The expression of pro- and anti-osteoclastogenic factors (*RANKL* and *OPG*, respectively) by the GALT model intestinal epithelial cells and PBMCs was quantified via RT-qPCR. Across four select donors, no significant differences were seen in OPG and RANKL expression across donor PBMCs as a function of SBD111 administration (Supplementary Fig. 5).

To characterize the contribution of SBD111-GALT interactions and SBD111 itself to osteoclastogenesis, RAW264.7 cells were treated with RANKL and conditioned basolateral supernatant from the GALT model with or without apically administered SBD111 (MOI −10 or 2), SBD111-conditioned supernatants (human cell free, equivalent MOI – 10 or 2; 6.67 × 10^5^ or 1.33 × 10^5^ CFU/mL, respectively), or media controls (Vehicle). Graphical representations of each GALT condition are indicated in Fig. 4A, while the workflow of this assay is illustrated in Fig. 4B. We observed that SBD111-treated GALT-conditioned basolateral media reduced TRAP activity in a SBD111 concentration-dependent manner (Fig. 4C). SBD111-conditioned media alone, without whole microbes or GALT-derived factors, induced a similar concentration-dependent reduction in osteoclast development. These data indicate that SBD111 secretes components/factors that reduce osteoclastogenesis.

**Fig. 5.**
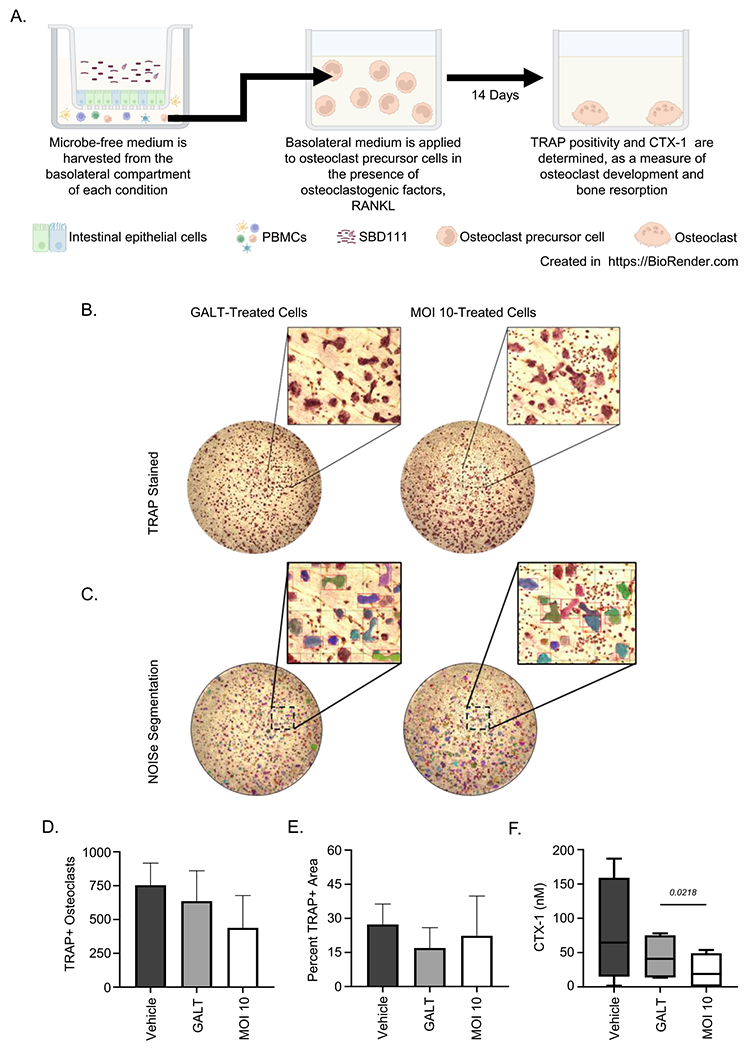
SBD111-treated media reduces the resorptive activity of osteoclasts derived from human PBMCs. **(A)** A pictographic illustration of the workflow for this experiment, created in BioRender. Green, R. (2025) https://BioRender.com/seaeapz. Osteoclasts derived from Human PBMCs were grown on bone chips in osteoclastogenic media for 14 days with media collected at day 12 and cells were fixed and stained for TRAP at day 14. Media treatments included GALT model media (Vehicle), media control-treated GALT-conditioned basolateral media (GALT), or apical SBD111-treated GALT-conditioned basolateral media (MOI 10). Following fixation, cells were stained for TRAP and quantified using NOISe. (**B**) Representative images of TRAP+ osteoclasts grown on bone chips illustrating effects of treated media. (**C**) Example of NOISe quantification and segmentation abilities. (**D**) NOISe-based quantification of osteoclast number and (**E**) TRAP+ area. (**F**) CTX-1 levels (a soluble measure of resorbed bone) were measured by ELISA in media collected from the osteoclast culture 12 days post isolation. Bars indicate mean and SEM based on *n* = 3 for count and area measures (**D, E**). Boxplots represent median and interquartile range, with whiskers representing max and min values, of *n* = 4 independent experiments for bone resorption (**F**). Vehicle condition is shown to display the normal range of human PBMC-derived osteoclast resorption and differentiation measures. Significance between conditions was determined by one-way ANOVA with Tukey’s HSD and significant *p* values (*p* < 0.05) are included presented.

Finally, to examine whether SBD111-GALT interactions result in factors that can inhibit bone resorption, Vehicle, GALT, and SBD111-treated GALT-conditioned (MOI 10) basolateral supernatants were added to cultures of primary human osteoclasts isolated from postmenopausal female donors and grown on bone slices for 14 days, as illustrated in Fig. 5A. The number of TRAP+ osteoclasts and the total TRAP+ area on each bone slice (Fig. 5B, C, D, and E) was not significantly different among the conditions. However, the MOI 10 supernatant significantly reduced media CTX-1, a bone resorption marker, levels compared to basolateral GALT supernatant alone (Fig. 5F), indicating MOI 10 apically-treated GALT basolateral media suppresses bone resorption without affecting osteoclast viability or differentiation.

## 4. Discussion

Osteopenia and osteoporosis commonly affect postmenopausal women and lead to increased fracture risk (Greendale et al., 2012; Reid & McClung, 2024). Options to mitigate bone loss are limited, and while pharmaceutical treatments exist, severe side effects limit their adoption until significant bone loss has occurred (Khan et al., 2023; MacLennan et al., 2004; Migliorati et al., 2010). To address this need, SBD111, a synbiotic medical food for the dietary management of postmenopausal bone loss was developed. In a recent double-blind, placebo-controlled clinical trial that enrolled women within 6 years of menopause, oral administration of SBD111 for one-year reduced bone loss in participants with osteopenia, elevated BMI, or elevated body fat (Schott et al., 2025). Given that, SBD111 administration also corresponded with reduced serum CTX-1 in participants with BMI ≥30 and the association between elevated BMI/body fat, gut barrier dysfunction, and systemic inflammation, these findings suggest that SBD111 exerts benefits on bone health by reducing inflammation and bone turnover. Here, we endeavored to determine potential mechanisms through which SBD111 impacts systemic bone health.

SBD111 was shown to reduce severe GI symptoms in early postmenopausal women, suggesting direct action in the gut. As such, we hypothesized that this synbiotic influences bone turnover through modulation of the intestinal barrier, a mechanism through which gastrointestinal microbes elicit broad systemic effects on immune function and health (Di Vincenzo et al., 2023a; Escalante et al., 2024). To test this, we examined this synbiotic’s effect on intestinal epithelial monolayers (Belguesmia et al., 2019; Ferraretto et al., 2018). We observed that SBD111 administration improved intestinal barrier functionality without inducing inflammatory chemokine secretion (IL-8 and CXCL1), independent of concentration. These phenotypes are consistent with previous reports that oral administration of beneficial microbes can reduce intestinal permeability, thereby limiting translocation of GI-resident microbes into surrounding tissues. This effect decreases local immune cell activation/inflammation and has been shown to reduce bone loss (Di Vincenzo et al., 2023a; Escalante et al., 2024; Thoo et al., 2019). Given that osteopenia and high BMI/visceral body fat are known to increase intestinal permeability and bacterial translocation, improvement in intestinal permeability may be one driver of the bone-sparing effects of SBD111 in those populations (Ferbebouh et al., 2021; Festa et al., 2001; Kredel & Siegmund, 2014; Teixeira et al., 2012; Zhang et al., 2022).

Although intestinal epithelial monolayers represent a common barrier model, they lack immune, stromal, and microbiome components (Belguesmia et al., 2019; Ferraretto et al., 2018). As such, these conclusions could be strengthened by examining SBD111’s effects on damaged epithelial membranes to model the leaky gut, common to high BMI/body fat individuals. Furthermore, future in vivo studies could confirm this mechanism through quantification of serum LPS or zonulin, as markers of intestinal permeability (Schoultz & Keita, 2020).

The menopause transition, obesity, and aging are all associated with increased inflammatory responses that can exacerbate bone loss (Festa et al., 2001; Kredel & Siegmund, 2014; Shieh et al., 2020; Thevaranjan et al., 2017). It has been proposed that these inflammatory responses are due, in part, to reduced intestinal barrier function, leading to local inflammation and immune activation within the gut (Di Vincenzo et al., 2023a; Thoo et al., 2019; Zaiss et al., 2019). Given that SBD111 reduced bone loss in a high BMI/body fat population, conditions which are associated with systemic inflammation, we hypothesized that in the context of the increased intestinal permeability common to these conditions, SBD111 administration may modulate the inflammatory responses of local immune cell populations within the gut, influencing systemic effects. To test this, we examined PBMC responses to SBD111 with or without the addition of an inflammatory challenge (LPS); to mimic the inflammatory conditions associated with osteopenia and high BMI/body fat-mediated barrier disruption. While PBMC-based techniques are limited by a lack of some GI-specific immune cell types, they are widely used to examine immune-probiotic interactions due to their complex cell populations and the ability to examine responses across many donors (Belguesmia et al., 2019; Hidalgo-Cantabrana et al., 2018; Kleiveland, 2015a; Sornkayasit et al., 2024).

In the absence of LPS challenge, low SBD111 concentrations induced PBMC secretion of cytokines that drive T-cell differentiation/maintenance and inflammation (IL-23 and IFN-γ) as well as immune cell migration (CXCL1), but production of these cytokines by PBMCs decreased with higher SBD111 concentrations (Cui et al., 2025; Muranski & Restifo, 2013; Pollard et al., 2013). Given that PBMCs defend the body from microbes, elevated cytokine production following introduction of microbes to naïve PBMCs is not an unexpected result, and this is commonly observed for probiotics (Hua et al., 2010; Ren et al., 2019).

In the context of an inflammatory challenge, SBD111 elicited a similar concentration-dependent response from PBMCs for IFN-γ and IL-23 in females. IL-6, CXCL1, and IL-8 exhibited strong, SBD111 concentration-dependent reductions in secretion by PBMCs following inflammatory challenge. These affected cytokines are inflammatory mediators that can drive inflammation and tissue damage, and importantly, these cytokines have been shown to directly (e.g., IL-23, IL-6, and IL-8) and indirectly (e.g., CXCL1 and IFN-γ) enhance osteoclastogenesis (Cai et al., 2024; Capucetti et al., 2020; Gao et al., 2006; Rizo-Téllez & Filep, 2024; Umur et al., 2024; Wang et al., 2025). Collectively, these observations suggest that SBD111 administration reduces cytokine secretion by immune cells. However, SBD111 has not been observed to alter serum cytokine responses in preclinical or human efficacy studies, suggesting that different local effects, such as the polarization and migration of immune cells, may drive SBD111s beneficial effects. It is well known that activated GI immune cells migrate throughout the body, eliciting distal effects (Di Vincenzo et al., 2023b; Galván-Peña et al., 2024). Consistently, SBD111 reduced cytokines that are critical for the polarization/maintenance of inflammatory T cells (IL-6, IFN-γ, and IL-23, non-significantly) and immune cell migration (IL-8 and CXCL1) under inflammatory challenge. As T cell polarization is known to affect osteoclast function, this intriguing possibility warrants further investigation in preclinical studies examining immune cell activation states within GI tissues and in circulating immune cell populations (Gao et al., 2006; Sato et al., 2006). Additionally, this observed dose dependency could be regulated temporally, wherein low doses may mirror high doses over an increased time scale. As such, future studies will examine these responses over a time course to determine whether a temporal factor is involved.

While SBD111 elicited anti-inflammatory responses after inflammatory challenge but not in the absence of inflammation, mirroring clinical results, this PBMC-based model lacks the gut microenvironment and epithelial cell protection. As such, these results are most relevant in the context of compromised intestinal barriers. Previously, we showed that SBD111 exhibits bone sparing effects in high BMI/body fat individuals but not in low BMI/body fat individuals (Schott et al., 2025). Given the relationship between visceral body fat and systemic inflammation, these data indicate that SBD111 may have an anti-inflammatory effect in those, and other systemically inflamed populations (Festa et al., 2001). However, based on these results, it would be beneficial to explore the effects of SBD111 on other populations. To this end, future experiments could source PBMCs from donors with different immunological backgrounds (e.g., those with other autoimmune/inflammatory disorders) to determine whether other inflamed populations would benefit from SBD111 administration, similar to the high BMI/body fat population examined clinically.

Additionally, we observed donor-dependent variation in PBMC responses to SBD111. One major source of this variation was sex. SBD111 administration, in the context of inflammatory challenge, significantly decreased CXCL1, IL-8, and IL-6 secretion by PBMCs of both sexes, while IFN-γ and IL-23 secretion was only significantly reduced for those of females. This could be due to sex-associated differences in immune cell populations. For example, it is well established that females have increased T cell populations and different dendritic cell activity relative to males (Chi et al., 2024; Hoffmann et al., 2023; Scotland et al., 2011). These differences in immune cell profiles could result in more pronounced changes in IL-23 and IFN-γ secretion for female donors, as these cytokines are primarily secreted by dendritic cells and T cells, respectively (Cui et al., 2025; Pollard et al., 2013). These data suggest that SBD111 stimulates particular cell types. Since specific immune cell classes play distinct roles in osteoclast development and activity, these interactions could be investigated more thoroughly through flow cytometric or single cell RNAseq methods to define which populations are activated by SBD111, characterize their cytokine production profiles, and determine whether there are population level changes (Sato et al., 2006; Tyagi et al., 2018; Wang et al., 2025). These results suggest that males may see similar, but muted benefits to SBD111 administration. Given that osteoporosis in men is becoming increasingly acknowledged as an unmet need associated with aging, the efficacy of SBD111 in male populations should also be explored in future clinical studies (Bandeira et al., 2022).

SBD111 was shown to reduce serum CTX-1 levels, a marker of bone degradation, in postmenopausal women with BMI ≥30, indicating that it likely inhibits osteoclast development or functioning, a process that is influenced by microbe-, epithelium-, and immune cell-derived molecules (Cai et al., 2024; Schott et al., 2025; Umur et al., 2024; Wang et al., 2025; Zaiss et al., 2019). To determine whether SBD111’s interactions in the gut and its resulting products underlie its bone sparing effects, PBMCs were cultured in the basolateral chamber of an in vitro gut model with apical administration of SBD111 (depicted in Fig. 4A and Fig. 4B). This is a complex multi-cellular model that contains many, but not all, of the pertinent cell types found in immunological tissues of the gut, resulting in a more physiologically relevant model capable of producing more complex responses. The SBD111-treated PBMC-conditioned media (basolateral supernatant representing the lamina propria) produced from this model was then incubated with murine RAW264.7 or human osteoclast precursor cells in the presence of osteoclast differentiating factors. Using this system, we determined that SBD111-treated basolateral supernatants reduced TRAP activity in RAW264.7 osteoclast precursor cells, indicating decreased osteoclast differentiation and/or function. Additionally, we showed that SBD111-derived factors directly inhibited TRAP activity by incubating RAW264.7 cells with SBD111-conditioned media without human cells. Similarly, we found that SBD111-conditioned PBMC supernatant administration also reduced a marker of bone resorption (CTX-1) in a more physiologically relevant in vitro model of human osteoclastogenesis and bone turnover. However, in this human primary cell model, the total number of osteoclasts (quantity of TRAP+ cells) was not affected, suggesting that SBD111 reduces osteoclast activity but not osteoclastogenesis. These observations are consistent with clinical data demonstrating that dietary intervention with SBD111 decreased serum CTX-1 in women with elevated BMI (Schott et al., 2025). While this model corroborates what is seen clinically, it does not necessarily reflect the local concentration of these products within bone which could affect the degree of inhibition seen in vivo.

While this study indicates potential mechanisms through which SBD111 may reduce bone loss, the models used have limitations. This work utilizes in vitro cell models, aligned with clinical and preclinical outcomes, to explore potential mechanisms of action that underlie SBD111s bone sparing benefits. Although this strategy allows for the study of a breadth of mechanisms, each model lacks components of the in vivo microenvironment, including the intestinal microbiome, gut specific immune cells, and additional bone cells (osteocytes and osteoblasts) that may interact with and respond to SBD111, its products, and the model systems. These interactions could be examined in future work through additional testing in vivo. Such future directions could include ovariectomized mouse models as previously utilized (Lawenius et al., 2022) with additional outputs including fecal metagenomic/metabolomic examination of changes in microbiome composition and function, serum zonulin/LPS quantification to examine changes in barrier integrity, RNAseq of intestinal tissues and bones to determine changes in local barrier, immune, and bone cell responses to SBD111 administration.

Another important limitation of this work relates to the specific metabolites and signaling pathways that underlie SBD111’s clinical effects. While it has previously been shown that SBD111 synergistically produces acetate, an anti-inflammatory compound, and has the capacity to produce Vitamin K_2_, a compound that positively influences bone health, this study focused only on the effects of SBD111 as a synbiotic consortium. As such, the contributions of specific strains and metabolites were not examined. Future work would benefit from investigation of all potentially immunomodulatory factors produced by SBD111, the signaling pathways that they activate, and the specific cell types impacted, both in terms of activation/memory state and population changes. As osteoclast development and activity have been shown to be regulated metabolically and immunologically, examining all signaling pathways is vital to further understanding SBD111’s mechanism of action (Cai et al., 2024; Ledesma-Colunga et al., 2023; Liang et al., 2025; Umur et al., 2024; Wang et al., 2025; Yu et al., 2025). Additionally, in vitro and in vivo examination of the contribution of each individual SBD111 strain would allow for optimization of future SBD111 formulations.

**Fig. 6.**
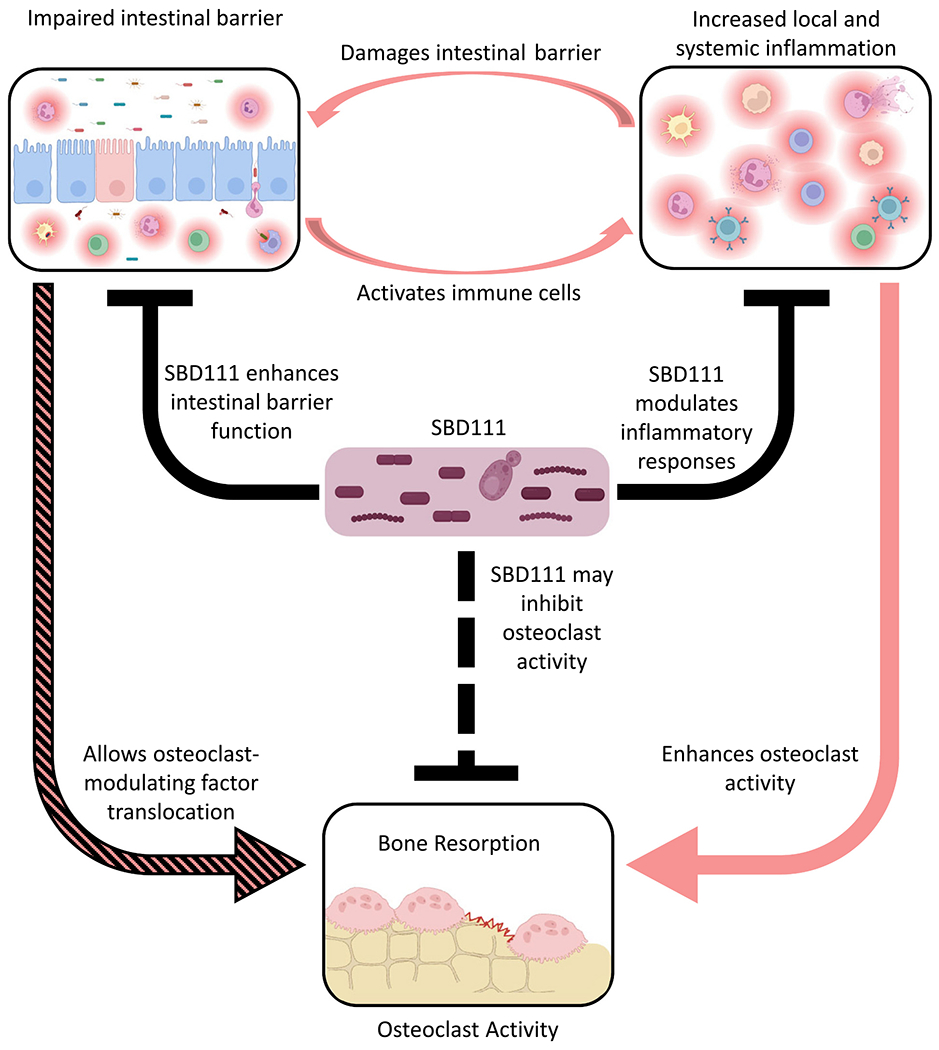
SBD111 interrupts the cycle of intestinal barrier disruption and systemic inflammation to inhibit osteoclast activity. Impaired intestinal barrier integrity, as occurs during aging, obesity, and the menopause transition permits translocation of inflammatory microbial components into intestinal tissues. This activates the epithelium and local immune cells which further compromise barrier integrity, allowing translocation of additional inflammatory components. This cycle promotes systemic inflammation and promotes osteoclastogenesis and bone resorption through the migration of osteoclast-activating inflammatory immune cells and diffusion of osteoclast-modulating compounds to bone. SBD111 disrupts this cycle by enhancing intestinal barrier integrity and, in the case of disrupted barriers, by reducing inflammatory mediator secretion (T cell polarizing and neutrophil chemoattractant) by inflamed immune cells. Furthermore, SBD111 has shown the potential to produce an anti-osteoclastogenic environment through interactions with intestinal epithelial cells and underlying immune cells. Together these mechanisms may contribute to the bone-protective effects of SBD111 observed in groups of postmenopausal women. Image created in https://BioRender.com. Green, R. (2025) https://BioRender.com/sty1qzs.

As summarized in Fig. 6, these data describe multifactorial mechanisms through which SBD111 may confer a clinical benefit for the management of postmenopausal bone loss. The menopausal transition, increased body fat, and aging are associated with reduced intestinal barrier integrity and increased systemic inflammation. These conditions can form a cycle in which intestinal barrier disruption activates local immune cells, which further disrupt the intestinal barrier and lead to systemic inflammation, osteoclast activation, and increased bone resorption. Here we show that, in vitro, SBD111 improves intestinal epithelial barrier integrity and reduces inflammatory responses by immune cells that lack a functional epithelial barrier, targeting both facets of this cycle, potentially facilitating a return to homeostasis. These data mirror our previous findings that SBD111 supplementation reduces severe GI symptoms and inflammatory signaling within bone (Lawenius et al., 2022; Schott et al., 2025). In addition, SBD111 exposure induces a concentration-dependent anti-osteoclastogenic environment in an in vitro gut model, supporting our clinical findings that dietary intervention with SBD111 reduces bone degradation markers in women with BMI ≥30 (Schott et al., 2025). Together, these results provide a mechanistic foundation for further clinical investigation of the synbiotic, SBD111, in populations at risk for inflammation-associated bone loss.

## 5. Conclusions

SBD111, a synbiotic medical food composed of microbes from fruits and vegetables as well as prebiotic fibers, has been shown to slow bone loss in women with BMI ≥30, with body fat ≥40 %, or with osteopenia. Here, we rigorously tested SBD111 to elucidate potential mechanisms underlying its benefits for bone health. We have determined that SBD111 may function through multiple mechanisms, wherein it can reduce osteoclast activity (bone degradation) through reductions in intestinal permeability and local inflammation or potentially through the production of anti-osteoclastogenic metabolites.

## Supplementary Material

1

Supplemental File

## Figures and Tables

**Table 1:** PBMC donor information.

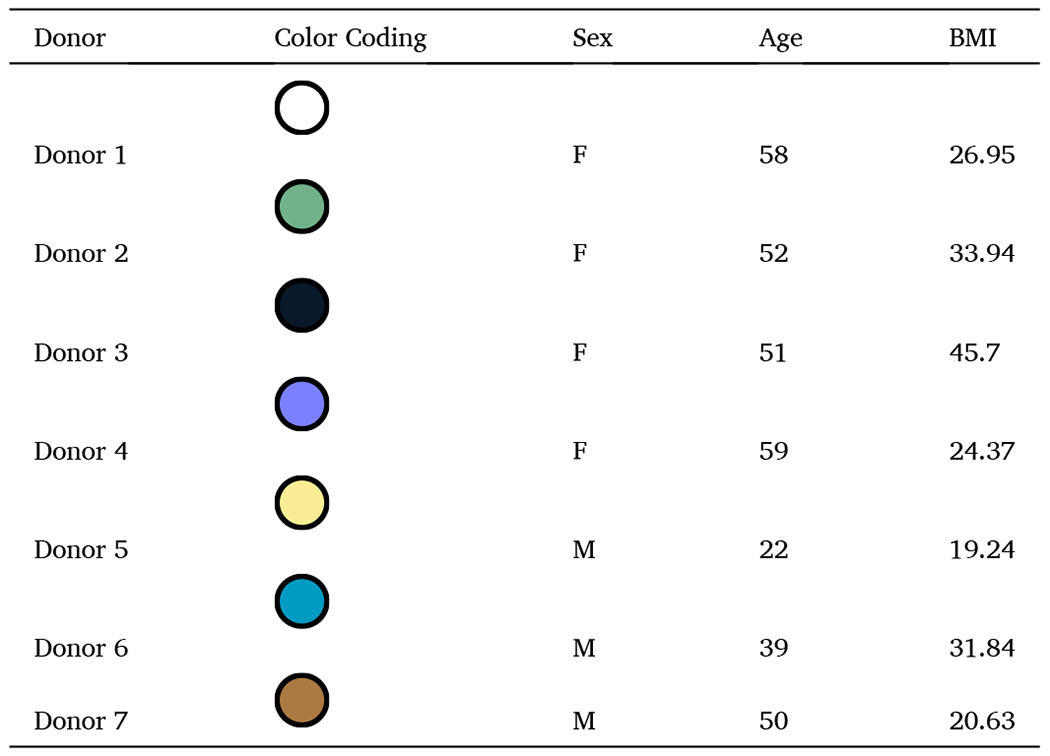

## Data Availability

The data described in the manuscript will be made available upon request.

## Appendix A. Supplementary data

Supplementary data to this article can be found online at https://doi.org/10.1016/j.jff.2025.107114.

## Abbreviations:

αMEM: alpha Minimal Essential Medium
ΔTEER: Change in Trans-epithelial Electrical Resistance
anti-anti: antibiotic-antimycotic
ATCC: American Type Culture Collection
BMD: Bone mineral density
CFU: Colony forming units
CTX-1: collagen cross-linked telopeptide
CXCL: C-X-C motif ligand
DMEM: Dulbecco’s Modified Eagle Medium
ELISA: Enzyme-Linked Immunosorbent Assay
FBS: Fetal Bovine Serum
GALT: gut-associated lymphoid tissue
GI: Gastrointestinal
IFN-γ: Interferon gamma
IL: Interleukin
M-CSF: Macrophage colony stimulating factor
MEM: Minimal Essential Medium
MOI: Multiplicity of interaction
OPG: Osteoprotegerin
PBMC: Peripheral blood mononuclear cells
PBS: Phosphate Buffered Saline
RANKL: Receptor activator of nuclear factor-κB Ligand
RPMI-1640: Roswell park Memorial Institute medium
TEER: Trans-epithelial Electrical Resistance
TNF-α: Tumor Necrosis factor alpha
TRAP: Tartrate-resistant acid phosphatase
TSB: Tryptic Soy Broth
