# Supplementary material for: A synbiotic medical food improves gut barrier function, reduces immune responses, and inhibits osteoclast activity in models of postmenopausal bone loss aligned with clinical outcomes
Source: J Funct Foods. Author manuscript; Available in PMC 2026 Jan 24. (PMC12829910; doi:10.1016/j.jff.2025.107114)
Supplement: Supplemental File [file NIHMS2130946-supplement-Supplemental_File.pptx]

## Slide 1
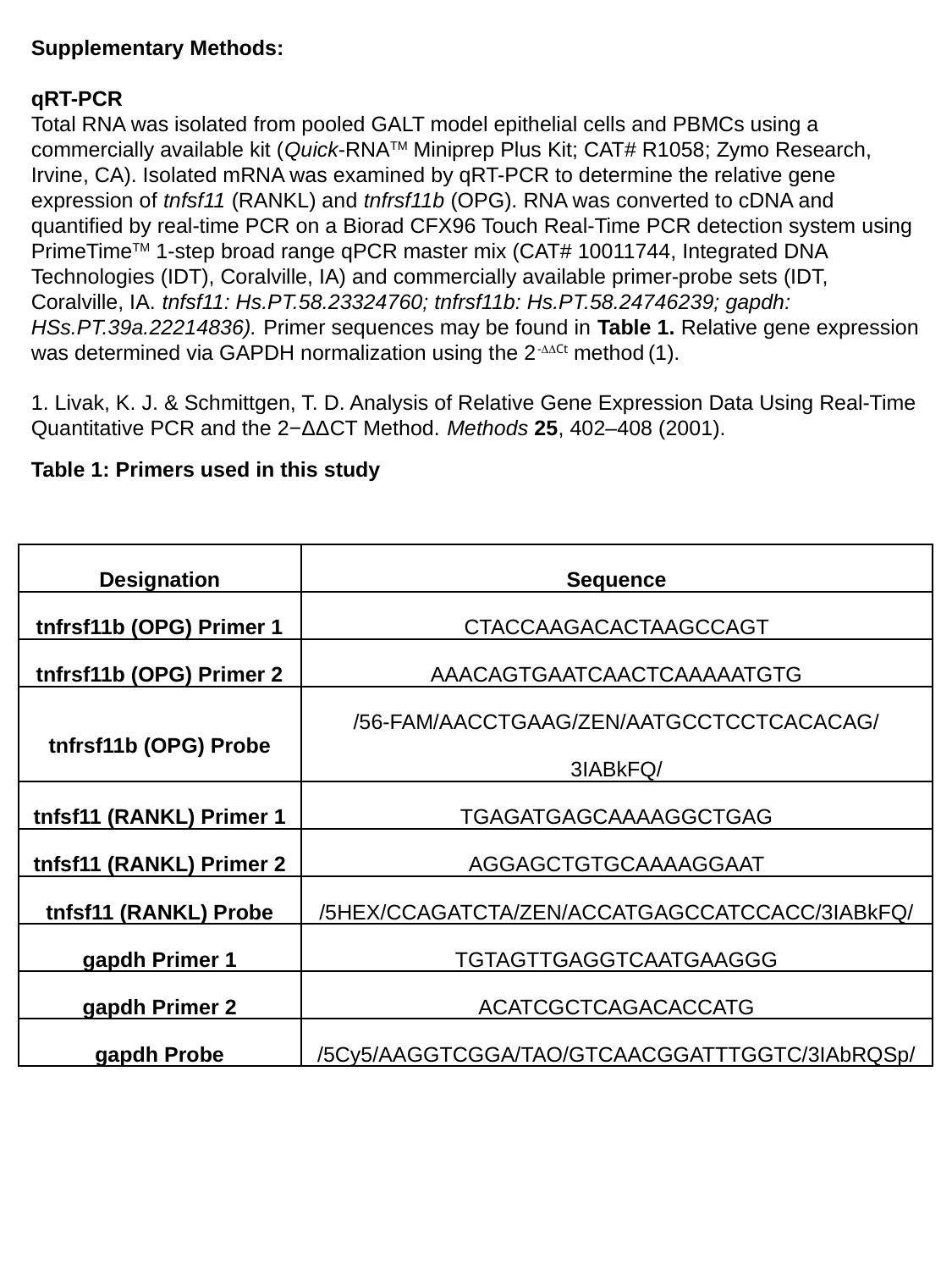

Supplementary Methods:
qRT-PCR
Total RNA was isolated from pooled GALT model epithelial cells and PBMCs using a commercially available kit (Quick-RNATM Miniprep Plus Kit; CAT# R1058; Zymo Research, Irvine, CA). Isolated mRNA was examined by qRT-PCR to determine the relative gene expression of tnfsf11 (RANKL) and tnfrsf11b (OPG). RNA was converted to cDNA and quantified by real-time PCR on a Biorad CFX96 Touch Real-Time PCR detection system using PrimeTimeTM 1-step broad range qPCR master mix (CAT# 10011744, Integrated DNA Technologies (IDT), Coralville, IA) and commercially available primer-probe sets (IDT, Coralville, IA. tnfsf11: Hs.PT.58.23324760; tnfrsf11b: Hs.PT.58.24746239; gapdh: HSs.PT.39a.22214836). Primer sequences may be found in Table 1. Relative gene expression was determined via GAPDH normalization using the 2-DDCt method (1).
1. Livak, K. J. & Schmittgen, T. D. Analysis of Relative Gene Expression Data Using Real-Time Quantitative PCR and the 2−ΔΔCT Method. Methods 25, 402–408 (2001).
Table 1: Primers used in this study
| Designation | Sequence |
| --- | --- |
| tnfrsf11b (OPG) Primer 1 | CTACCAAGACACTAAGCCAGT |
| tnfrsf11b (OPG) Primer 2 | AAACAGTGAATCAACTCAAAAATGTG |
| tnfrsf11b (OPG) Probe | /56-FAM/AACCTGAAG/ZEN/AATGCCTCCTCACACAG/3IABkFQ/ |
| tnfsf11 (RANKL) Primer 1 | TGAGATGAGCAAAAGGCTGAG |
| tnfsf11 (RANKL) Primer 2 | AGGAGCTGTGCAAAAGGAAT |
| tnfsf11 (RANKL) Probe | /5HEX/CCAGATCTA/ZEN/ACCATGAGCCATCCACC/3IABkFQ/ |
| gapdh Primer 1 | TGTAGTTGAGGTCAATGAAGGG |
| gapdh Primer 2 | ACATCGCTCAGACACCATG |
| gapdh Probe | /5Cy5/AAGGTCGGA/TAO/GTCAACGGATTTGGTC/3IAbRQSp/ |

## Slide 2
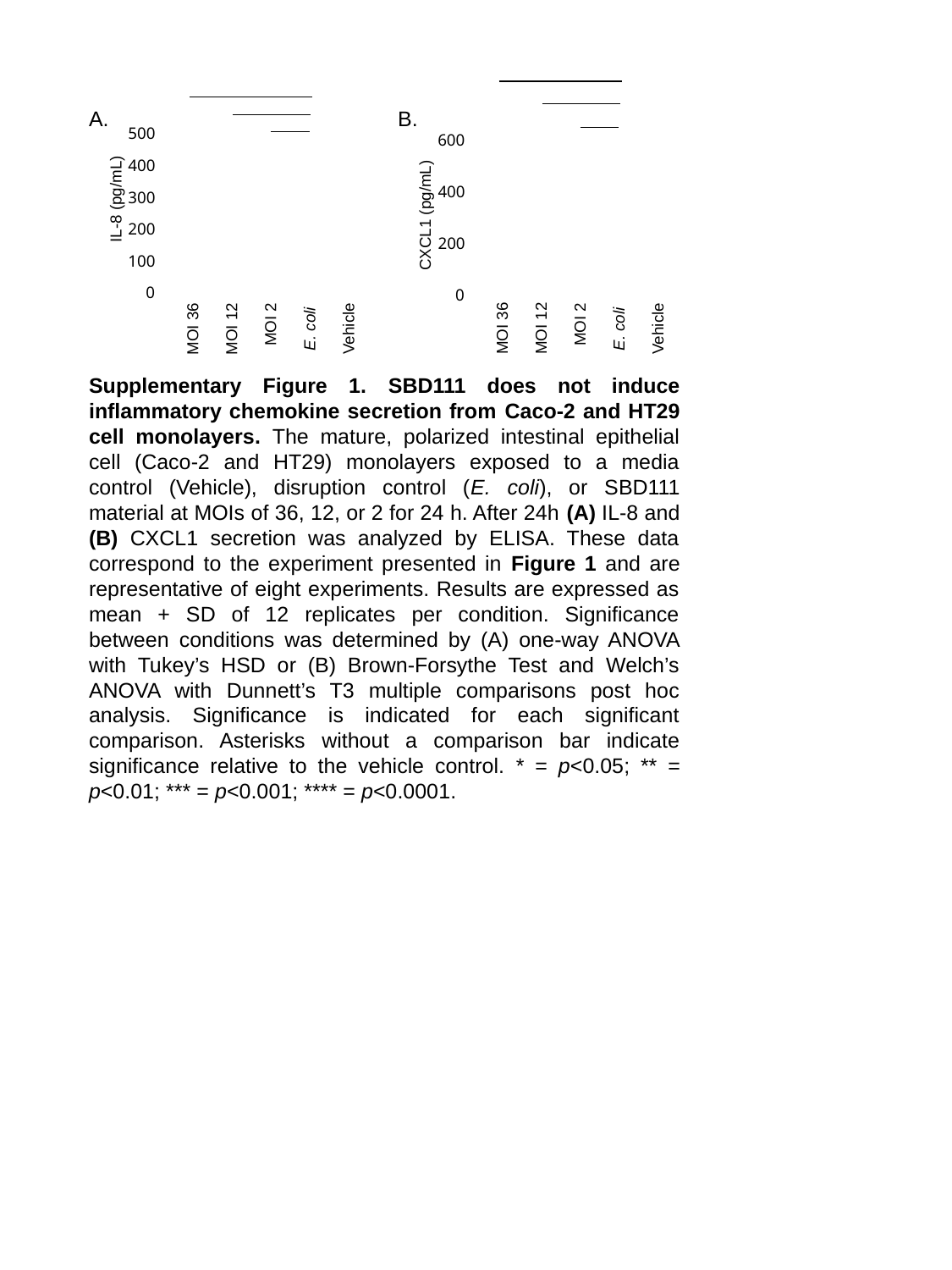

<0.0001
<0.0001
<0.0001
<0.0001
A.
B.
<0.0001
<0.0001
500
<0.0001
<0.0001
600
400
400
300
IL-8 (pg/mL)
CXCL1 (pg/mL)
200
200
100
0
0
MOI 36
MOI 12
MOI 2
E. coli
Vehicle
MOI 36
MOI 12
MOI 2
E. coli
Vehicle
Supplementary Figure 1. SBD111 does not induce inflammatory chemokine secretion from Caco-2 and HT29 cell monolayers. The mature, polarized intestinal epithelial cell (Caco-2 and HT29) monolayers exposed to a media control (Vehicle), disruption control (E. coli), or SBD111 material at MOIs of 36, 12, or 2 for 24 h. After 24h (A) IL-8 and (B) CXCL1 secretion was analyzed by ELISA. These data correspond to the experiment presented in Figure 1 and are representative of eight experiments. Results are expressed as mean + SD of 12 replicates per condition. Significance between conditions was determined by (A) one-way ANOVA with Tukey’s HSD or (B) Brown-Forsythe Test and Welch’s ANOVA with Dunnett’s T3 multiple comparisons post hoc analysis. Significance is indicated for each significant comparison. Asterisks without a comparison bar indicate significance relative to the vehicle control. * = p<0.05; ** = p<0.01; *** = p<0.001; **** = p<0.0001.

## Slide 3
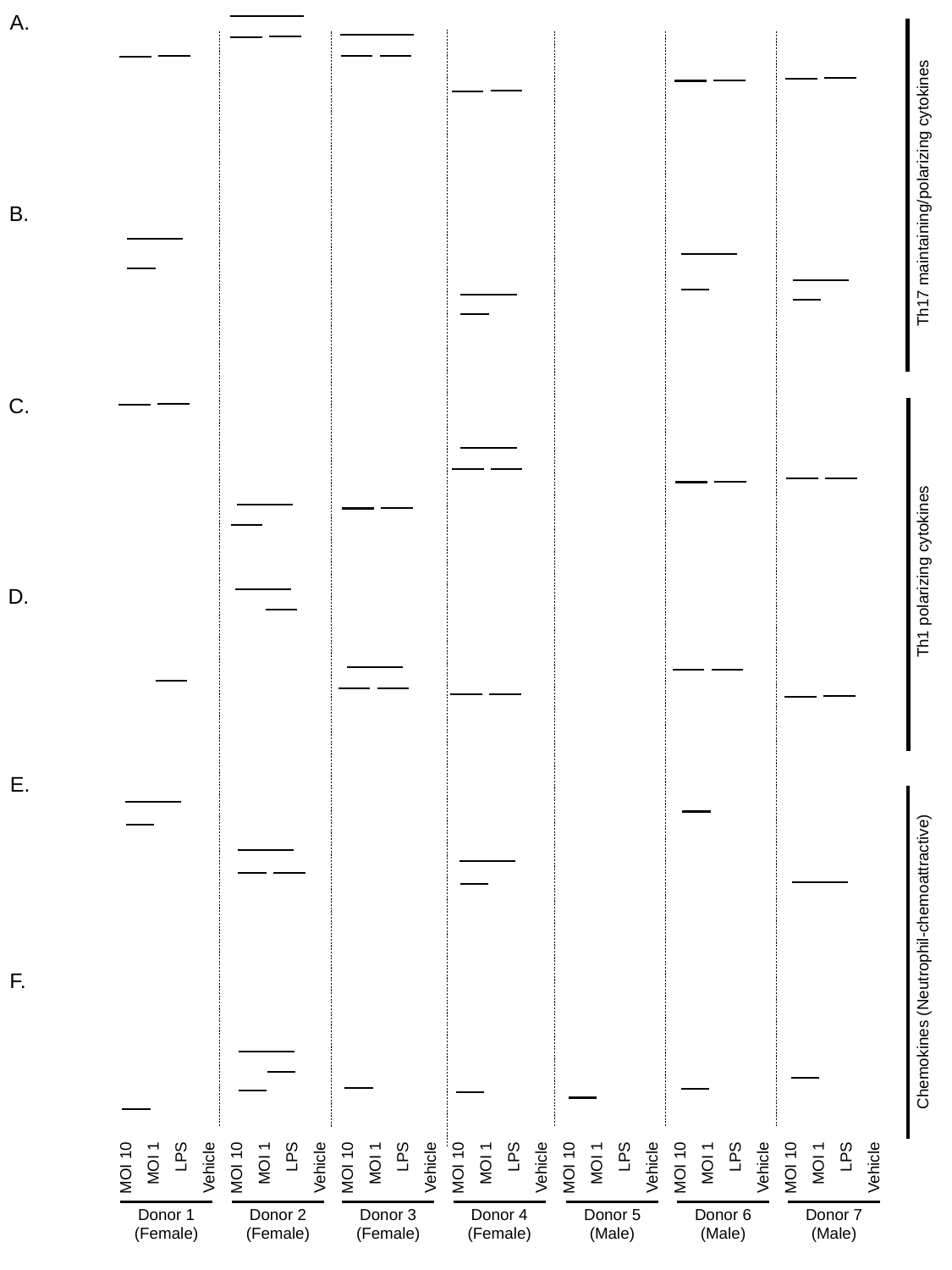

A.
 Th17 maintaining/polarizing cytokines
B.
C.
 Th1 polarizing cytokines
D.
E.
 Chemokines (Neutrophil-chemoattractive)
F.
MOI 10
MOI 1
LPS
Vehicle
MOI 10
MOI 1
LPS
Vehicle
MOI 10
MOI 1
LPS
Vehicle
MOI 10
MOI 1
LPS
Vehicle
MOI 10
MOI 1
LPS
Vehicle
MOI 10
MOI 1
LPS
Vehicle
MOI 10
MOI 1
LPS
Vehicle
Donor 1 (Female)
Donor 2 (Female)
Donor 3 (Female)
Donor 4 (Female)
Donor 5 (Male)
Donor 6 (Male)
Donor 7 (Male)

## Slide 4
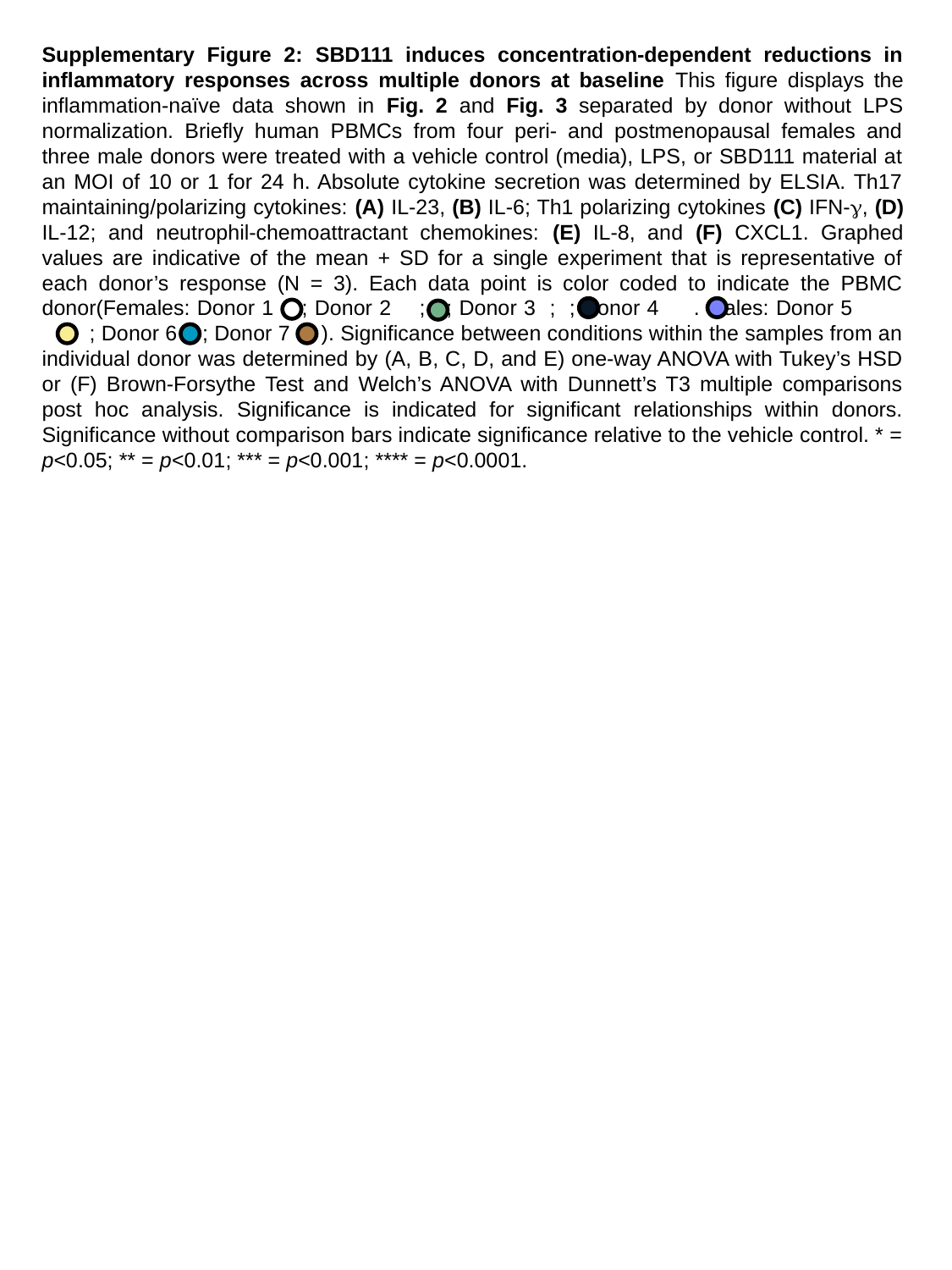

Supplementary Figure 2: SBD111 induces concentration-dependent reductions in inflammatory responses across multiple donors at baseline This figure displays the inflammation-naïve data shown in Fig. 2 and Fig. 3 separated by donor without LPS normalization. Briefly human PBMCs from four peri- and postmenopausal females and three male donors were treated with a vehicle control (media), LPS, or SBD111 material at an MOI of 10 or 1 for 24 h. Absolute cytokine secretion was determined by ELSIA. Th17 maintaining/polarizing cytokines: (A) IL-23, (B) IL-6; Th1 polarizing cytokines (C) IFN-g, (D) IL-12; and neutrophil-chemoattractant chemokines: (E) IL-8, and (F) CXCL1. Graphed values are indicative of the mean + SD for a single experiment that is representative of each donor’s response (N = 3). Each data point is color coded to indicate the PBMC donor(Females: Donor 1 ; Donor 2 ; ; Donor 3 ; ; Donor 4 . Males: Donor 5 dddd; Donor 6 ; Donor 7 ). Significance between conditions within the samples from an individual donor was determined by (A, B, C, D, and E) one-way ANOVA with Tukey’s HSD or (F) Brown-Forsythe Test and Welch’s ANOVA with Dunnett’s T3 multiple comparisons post hoc analysis. Significance is indicated for significant relationships within donors. Significance without comparison bars indicate significance relative to the vehicle control. * = p<0.05; ** = p<0.01; *** = p<0.001; **** = p<0.0001.

## Slide 5
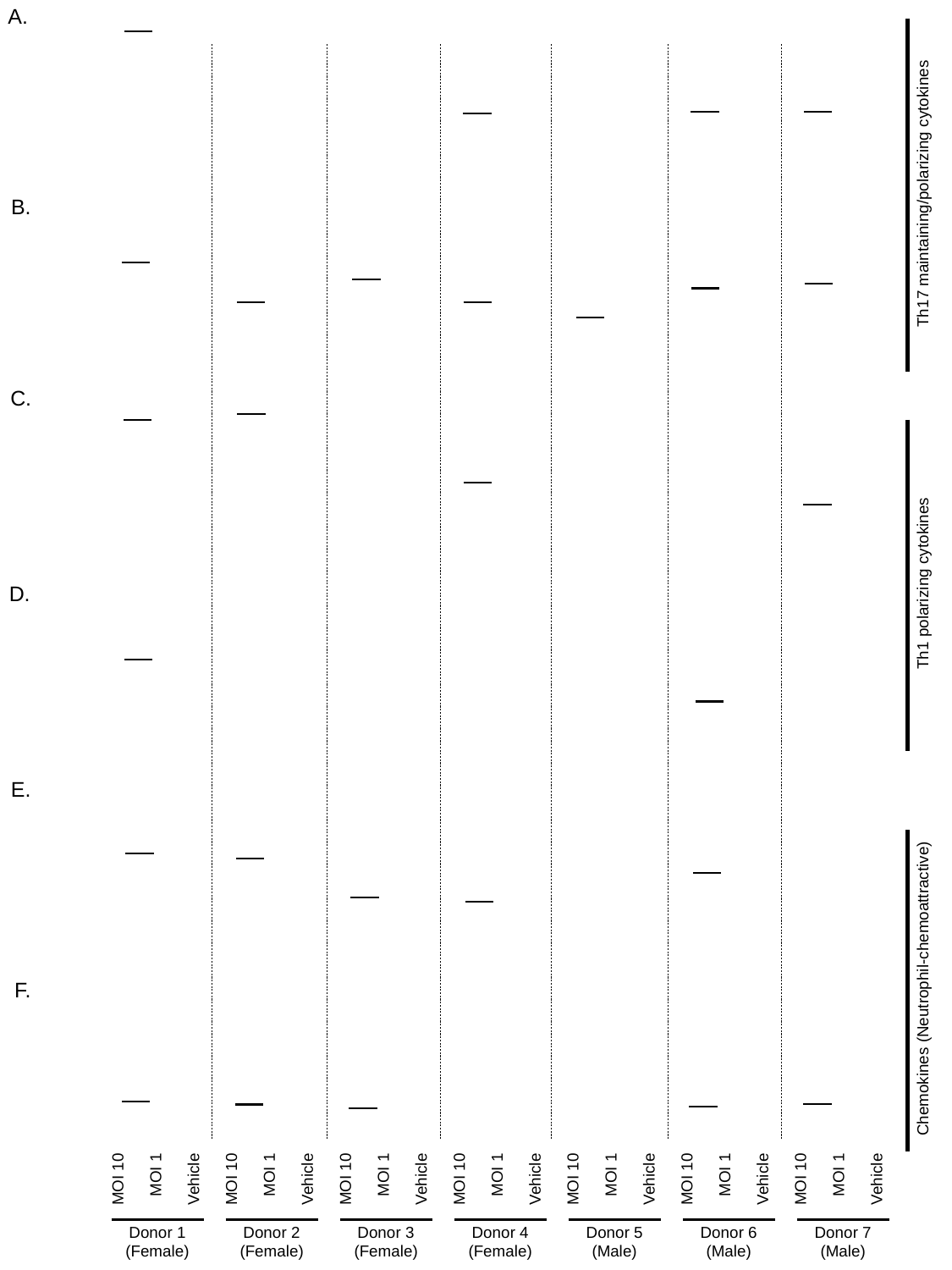

A.
 Th17 maintaining/polarizing cytokines
B.
C.
 Th1 polarizing cytokines
D.
E.
F.
MOI 10
MOI 1
Vehicle
MOI 10
MOI 1
Vehicle
MOI 10
MOI 1
Vehicle
MOI 10
MOI 1
Vehicle
MOI 10
MOI 1
Vehicle
MOI 10
MOI 1
Vehicle
MOI 10
MOI 1
Vehicle
Donor 1 (Female)
Donor 2 (Female)
Donor 3 (Female)
Donor 4 (Female)
Donor 5 (Male)
Donor 6 (Male)
Donor 7 (Male)
 Chemokines (Neutrophil-chemoattractive)

## Slide 6
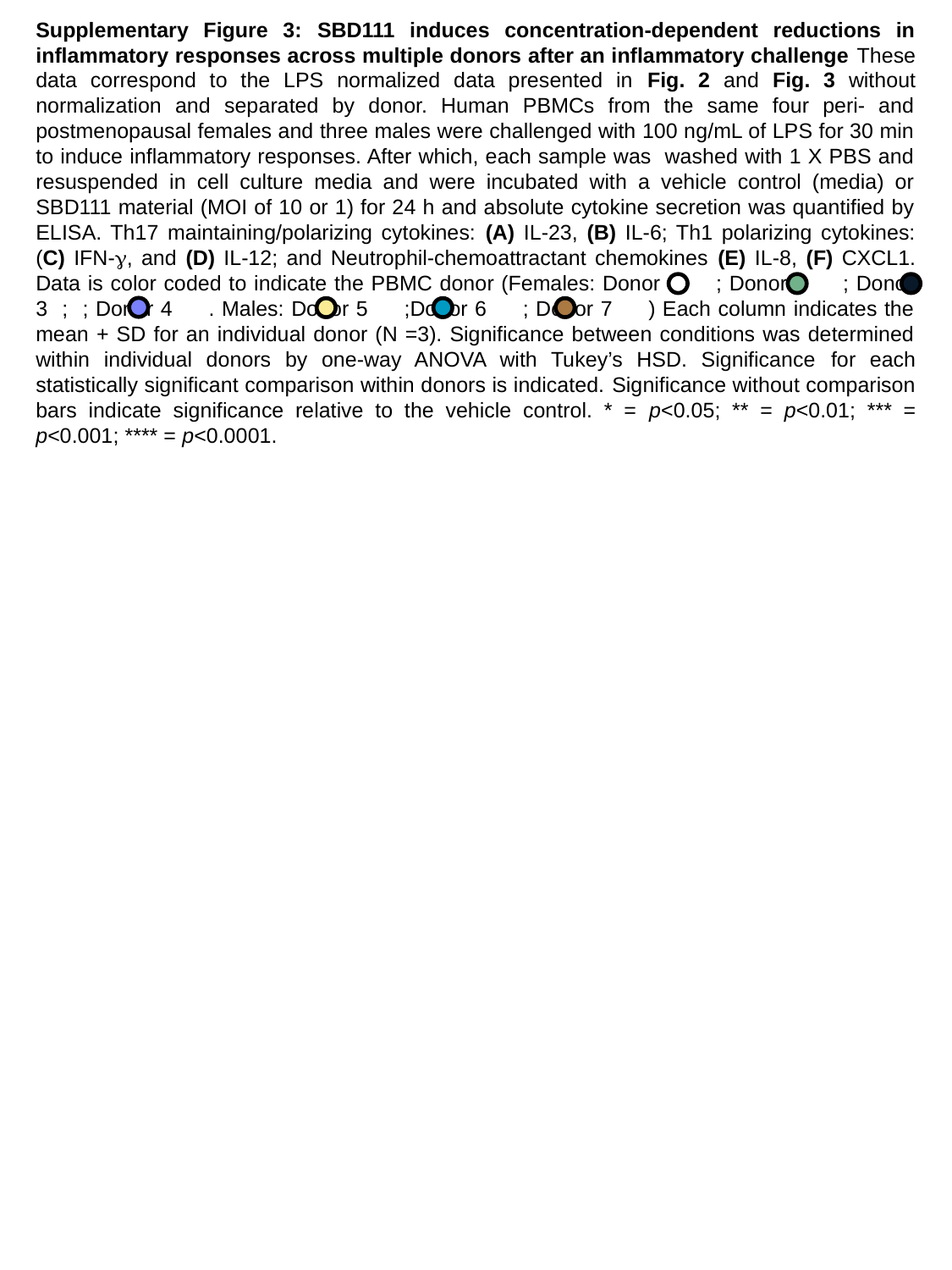

Supplementary Figure 3: SBD111 induces concentration-dependent reductions in inflammatory responses across multiple donors after an inflammatory challenge These data correspond to the LPS normalized data presented in Fig. 2 and Fig. 3 without normalization and separated by donor. Human PBMCs from the same four peri- and postmenopausal females and three males were challenged with 100 ng/mL of LPS for 30 min to induce inflammatory responses. After which, each sample was washed with 1 X PBS and resuspended in cell culture media and were incubated with a vehicle control (media) or SBD111 material (MOI of 10 or 1) for 24 h and absolute cytokine secretion was quantified by ELISA. Th17 maintaining/polarizing cytokines: (A) IL-23, (B) IL-6; Th1 polarizing cytokines: (C) IFN-g, and (D) IL-12; and Neutrophil-chemoattractant chemokines (E) IL-8, (F) CXCL1. Data is color coded to indicate the PBMC donor (Females: Donor 1 ; Donor 2 ; Donor 3 ; ; Donor 4 . Males: Donor 5 ;Donor 6 ; Donor 7 ) Each column indicates the mean + SD for an individual donor (N =3). Significance between conditions was determined within individual donors by one-way ANOVA with Tukey’s HSD. Significance for each statistically significant comparison within donors is indicated. Significance without comparison bars indicate significance relative to the vehicle control. * = p<0.05; ** = p<0.01; *** = p<0.001; **** = p<0.0001.

## Slide 7
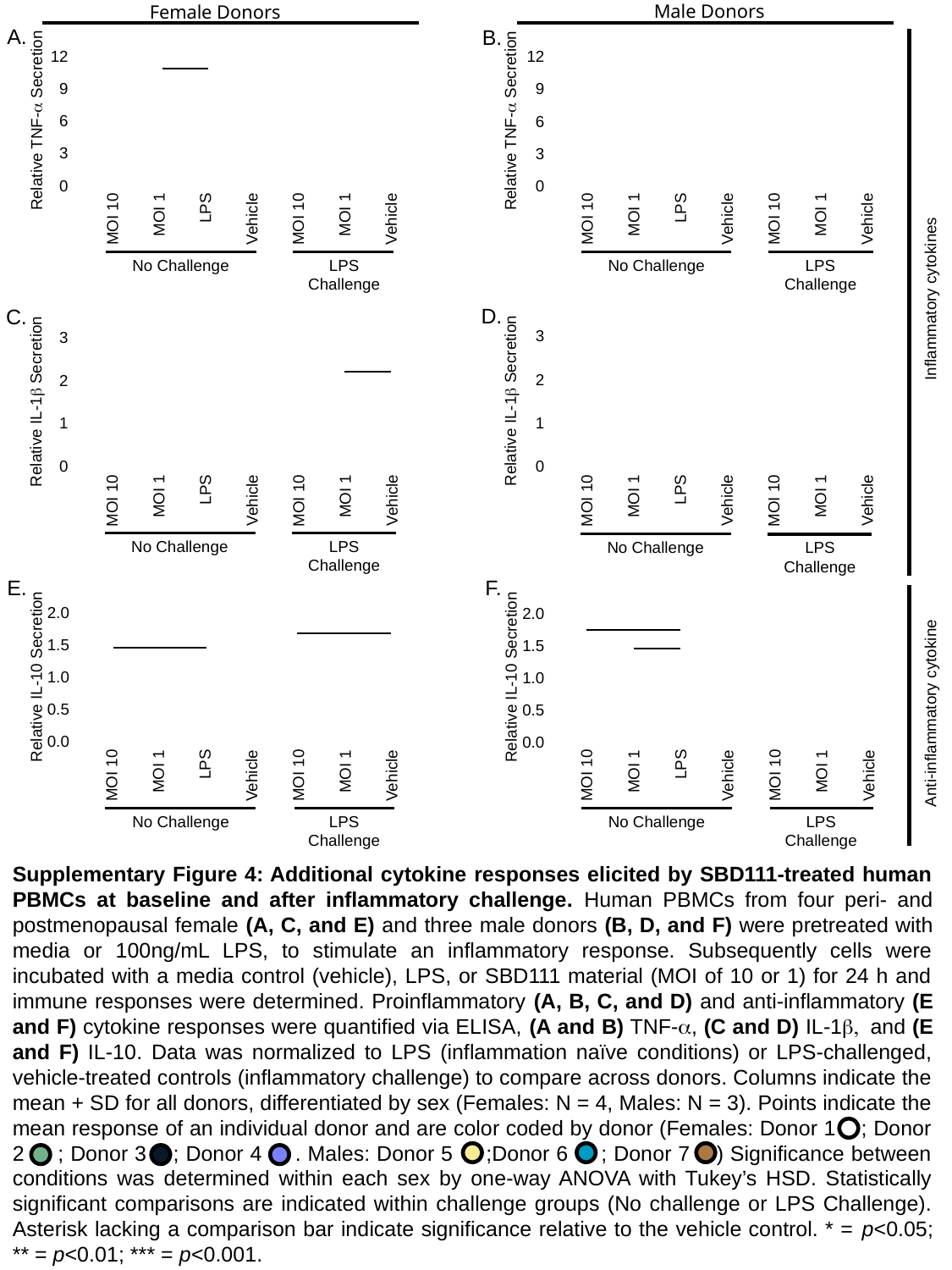

Male Donors
Female Donors
A.
12
9
Relative TNF-a Secretion
6
3
0
MOI 10
MOI 10
MOI 1
LPS
Vehicle
MOI 1
Vehicle
No Challenge
LPS Challenge
B.
12
9
Relative TNF-a Secretion
6
0
MOI 10
MOI 1
LPS
Vehicle
MOI 10
MOI 1
Vehicle
No Challenge
LPS Challenge
3
 Inflammatory cytokines
C.
3
2
Relative IL-1b Secretion
1
0
MOI 10
MOI 1
LPS
Vehicle
MOI 10
MOI 1
Vehicle
No Challenge
LPS Challenge
D.
3
2
Relative IL-1b Secretion
1
0
MOI 10
MOI 1
LPS
Vehicle
MOI 10
MOI 1
Vehicle
No Challenge
LPS Challenge
2.0
1.5
1.0
Relative IL-10 Secretion
0.0
E.
MOI 10
MOI 1
LPS
Vehicle
MOI 10
MOI 1
Vehicle
No Challenge
LPS Challenge
0.5
F.
2.0
1.5
Relative IL-10 Secretion
1.0
0.0
MOI 10
MOI 1
LPS
Vehicle
MOI 10
MOI 1
Vehicle
No Challenge
LPS Challenge
0.5
 Anti-inflammatory cytokine
Supplementary Figure 4: Additional cytokine responses elicited by SBD111-treated human PBMCs at baseline and after inflammatory challenge. Human PBMCs from four peri- and postmenopausal female (A, C, and E) and three male donors (B, D, and F) were pretreated with media or 100ng/mL LPS, to stimulate an inflammatory response. Subsequently cells were incubated with a media control (vehicle), LPS, or SBD111 material (MOI of 10 or 1) for 24 h and immune responses were determined. Proinflammatory (A, B, C, and D) and anti-inflammatory (E and F) cytokine responses were quantified via ELISA, (A and B) TNF-a, (C and D) IL-1b, and (E and F) IL-10. Data was normalized to LPS (inflammation naïve conditions) or LPS-challenged, vehicle-treated controls (inflammatory challenge) to compare across donors. Columns indicate the mean + SD for all donors, differentiated by sex (Females: N = 4, Males: N = 3). Points indicate the mean response of an individual donor and are color coded by donor (Females: Donor 1 ; Donor 2 ; Donor 3 ; Donor 4 . Males: Donor 5 ;Donor 6 ; Donor 7 ) Significance between conditions was determined within each sex by one-way ANOVA with Tukey’s HSD. Statistically significant comparisons are indicated within challenge groups (No challenge or LPS Challenge). Asterisk lacking a comparison bar indicate significance relative to the vehicle control. * = p<0.05; ** = p<0.01; *** = p<0.001.

## Slide 8
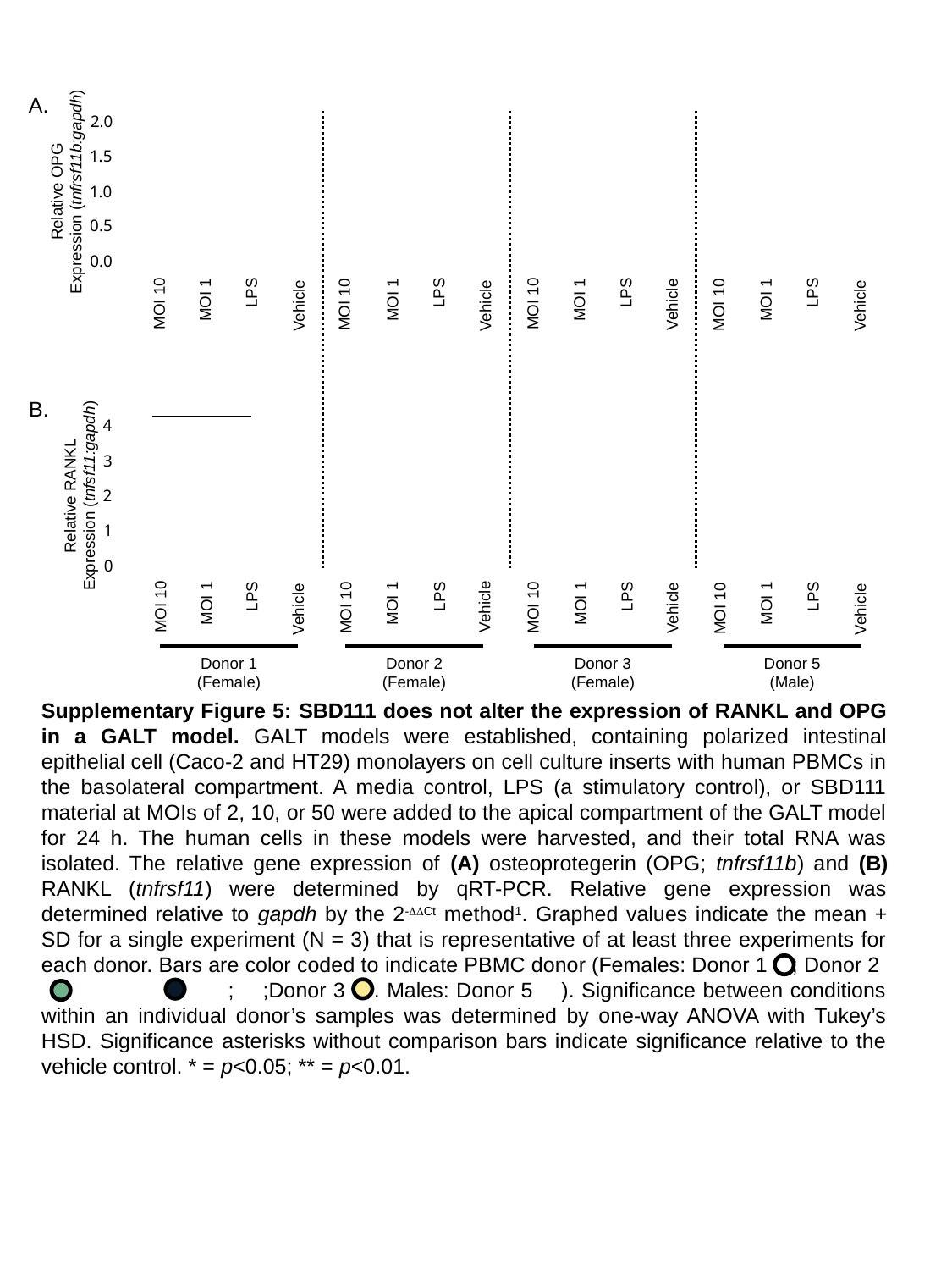

A.
2.0
1.5
Relative OPG
Expression (tnfrsf11b:gapdh)
1.0
0.5
0.0
LPS
LPS
LPS
LPS
MOI 1
MOI 1
MOI 1
MOI 1
MOI 10
MOI 10
Vehicle
MOI 10
MOI 10
Vehicle
Vehicle
Vehicle
B.
4
3
Relative RANKL
Expression (tnfsf11:gapdh)
2
1
0
LPS
LPS
LPS
LPS
MOI 1
MOI 1
MOI 1
MOI 1
MOI 10
MOI 10
Vehicle
MOI 10
MOI 10
Vehicle
Vehicle
Vehicle
Donor 1 (Female)
Donor 2 (Female)
Donor 3 (Female)
Donor 5 (Male)
Supplementary Figure 5: SBD111 does not alter the expression of RANKL and OPG in a GALT model. GALT models were established, containing polarized intestinal epithelial cell (Caco-2 and HT29) monolayers on cell culture inserts with human PBMCs in the basolateral compartment. A media control, LPS (a stimulatory control), or SBD111 material at MOIs of 2, 10, or 50 were added to the apical compartment of the GALT model for 24 h. The human cells in these models were harvested, and their total RNA was isolated. The relative gene expression of (A) osteoprotegerin (OPG; tnfrsf11b) and (B) RANKL (tnfrsf11) were determined by qRT-PCR. Relative gene expression was determined relative to gapdh by the 2-DDCt method1. Graphed values indicate the mean + SD for a single experiment (N = 3) that is representative of at least three experiments for each donor. Bars are color coded to indicate PBMC donor (Females: Donor 1 ; Donor 2 ; ;Donor 3 . Males: Donor 5 ). Significance between conditions within an individual donor’s samples was determined by one-way ANOVA with Tukey’s HSD. Significance asterisks without comparison bars indicate significance relative to the vehicle control. * = p<0.05; ** = p<0.01.
